# Gold(iii) complexes of 2-*tert*-butyl-1,10-phenanthroline and of *N*-(8-quinolinyl)amides: syntheses and structures

**DOI:** 10.1039/d6ra07844c

**Published:** 2026-09-07

**Authors:** David R. Weinberg, Kaitlyn M. Rutter, Joshua E. Thompson, Matt Sleck, Nicholas A. Curry, Alex E. Eckberg, Julian A. Manila, Hayden R. Murphy, Karina Saucedo Chavez, Siya R. Salunke, Chloe N. Angello, Zoe Steele, Arnold L. Rheingold

**Affiliations:** a Colorado Mesa University, Department of Physical and Environmental Sciences 1100 North Ave. Grand Junction CO 81501 USA dweinber@coloradomesa.edu; b University of California, San Diego, Department of Chemistry and Biochemistry 9500 Gilman Dr. La Jolla CA 92093 USA

## Abstract

A series of gold(iii) complexes was synthesized by mixing aqueous solutions of [AuCl_4_]^−^ with organic solutions of the appropriate ligand (2-*t*-butyl-1,10-phenanthroline (^*t*-Bu^Phen), 2,2-dimethyl-*N*-8-quinolinylpropanamide (^*t*-Bu^AQ), or 2-ethyl-*N*-8-quinolinylbutanamide (^3-Pent^AQ)). This resulted in the respective complexes [(^*t*-Bu^Phen)AuCl_3_], [(^*t*-Bu^AQ)AuCl_2_], and [(^3-Pent^AQ)AuCl_2_]. The formation of the latter two complexes involved deprotonation of the amide; however, the inclusion of NaHCO_3_ (a potential base) in the reaction mixture improved the yields of [(^*t*-Bu^Phen)AuCl_3_] and [(^*t*-Bu^AQ)AuCl_2_] but not of [(^3-Pent^AQ)AuCl_2_]. In assessing the crystal structure of [(^*t*-Bu^Phen)AuCl_3_], the three chlorides and the less sterically hindered nitrogen of the phenanthroline make a square plane around the gold(iii) center, while the more sterically hindered nitrogen of the phenanthroline is positioned to interact with the gold(iii) in an apical position. Any interaction between gold(iii) and the apical nitrogen is very weak based on their relatively large separation and the lack of much distortion in the square plane. The complexes [(^*t*-Bu^AQ)AuCl_2_] and [(^3-Pent^AQ)AuCl_2_] are both square planar with the organic ligand bound in a bidentate manner through imine and amido donors. In comparing the structures of all these complexes, it is apparent that bond lengths, angles, and degree of bonding are strongly impacted by the sterics associated with the presence of a *t*-butyl group near the gold(iii) center.

## Abstract 2

Three novel gold(iii) complexes containing either 1,10-phenanthroline- or 8-aminoquinoline-based ligands have been synthesized and analyzed for the impacts of sterics.

## Graphical abstract

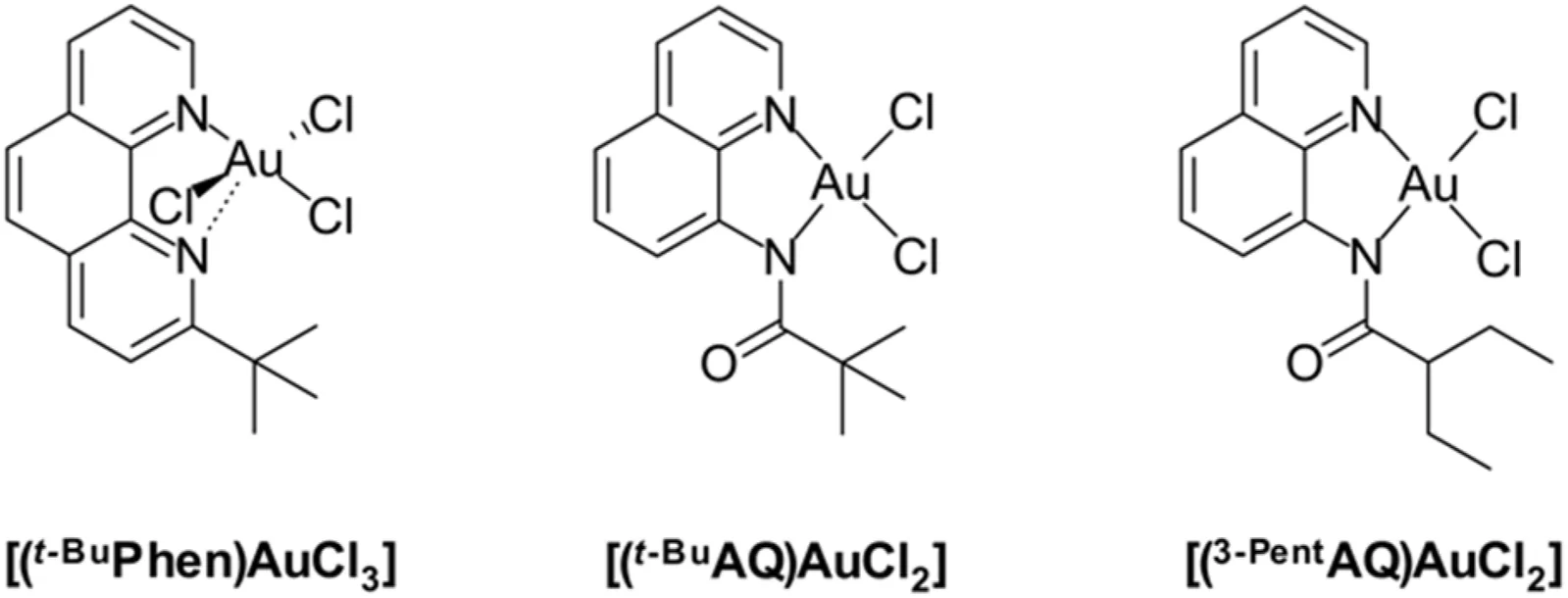

## Introduction

Gold(iii) complexes have shown potential both as anti-cancer drugs^1–3^ and as C–H bond functionalization catalysts.^4,5^ With respect to the former, gold(iii) compounds may prove complementary to popular platinum(ii)-based anti-cancer drugs as the gold(iii) complexes seem to generally operate by different mechanisms of action.^3^ Unfortunately, the development of gold(iii) anti-cancer drugs has been slowed by issues of stability and solubility under physiological conditions.^1,3^ Fortunately, gold(iii) stability, solubility, and even mechanism of action can be dramatically impacted by the specific choice of ligands and in particular by the steric bulk positioned near the metal-binding donors.^1–3,6^ Here, we examine this steric influence on gold binding modes and geometries for two distinct classes of bidentate *N*,*N*-donor ligands: substituted 1,10 phenanthrolines and amides of 8-aminoquinoline.

1,10-Phenanthroline (Phen) ligands are particularly useful for probing steric effects from groups adjacent to gold(iii)-binding donors, since they bind gold(iii) in distinct coordination modes depending on substitution at the 2- and 9-positions.^7–11^ Unsubstituted Phen tends to bind in a bidentate manner, forming the square planar cation [(Phen)AuCl_2_]^+^ (Fig. 1).^12^ Introducing a substituent at the 2-position (^R^Phen) or at both the 2- and 9-positions (^R2^Phen), however, favors a pseudo-five-coordinate neutral complex in which the phenanthroline ligand remains bound to gold(iii) alongside three chlorides, with one Au–N bond markedly elongated (Fig. 1).^7–11^ At the extreme, steric hindrance near the nitrogen donors has thus far prevented isolation of a gold(iii) complex with 2,9-di-*tert*-butyl-1,10-phenanthroline.^7^ Herein, we report the first synthesis of a gold(iii) complex bearing the mono-substituted analog, 2-*t*-butyl-1,10-phenanthroline (^*t*-Bu^Phen); this complex, [(^*t*-Bu^Phen)AuCl_3_], is depicted in Fig. 2.

**Fig. 1:**
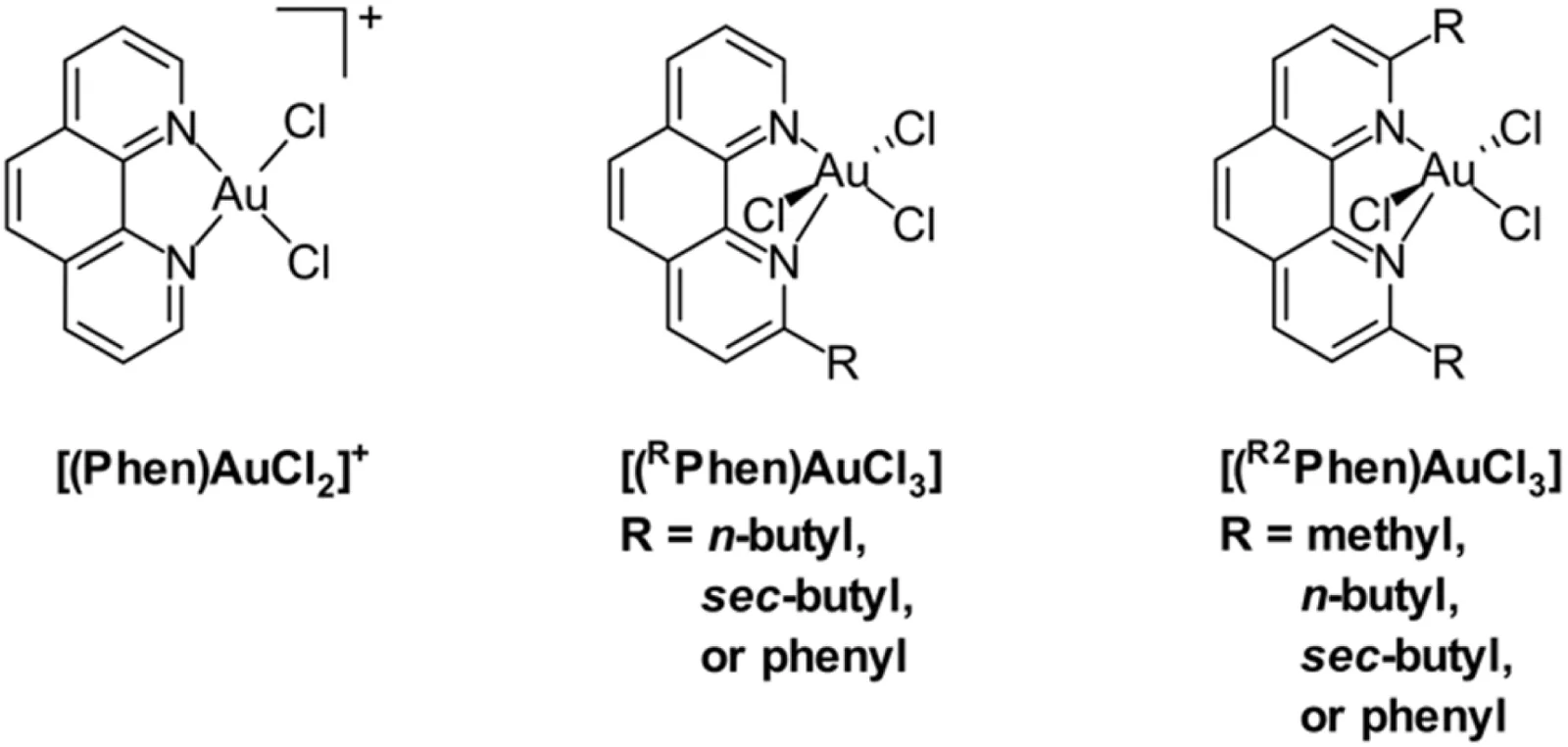
Binding modes of gold(iii) complexes with unsubstituted, 2-substituted, and 2,9-disubstituted 1,10-phenanthroline ligands. The [(Phen)AuCl_2_]^+^ cation is square planar. In contrast, each complex with 2-substituted or 2,9-disubstituted 1,10-phenanthroline ligands is pseudo five coordinate with one of the Au–N bonds being very elongated.^7–11^

**Fig. 2:**
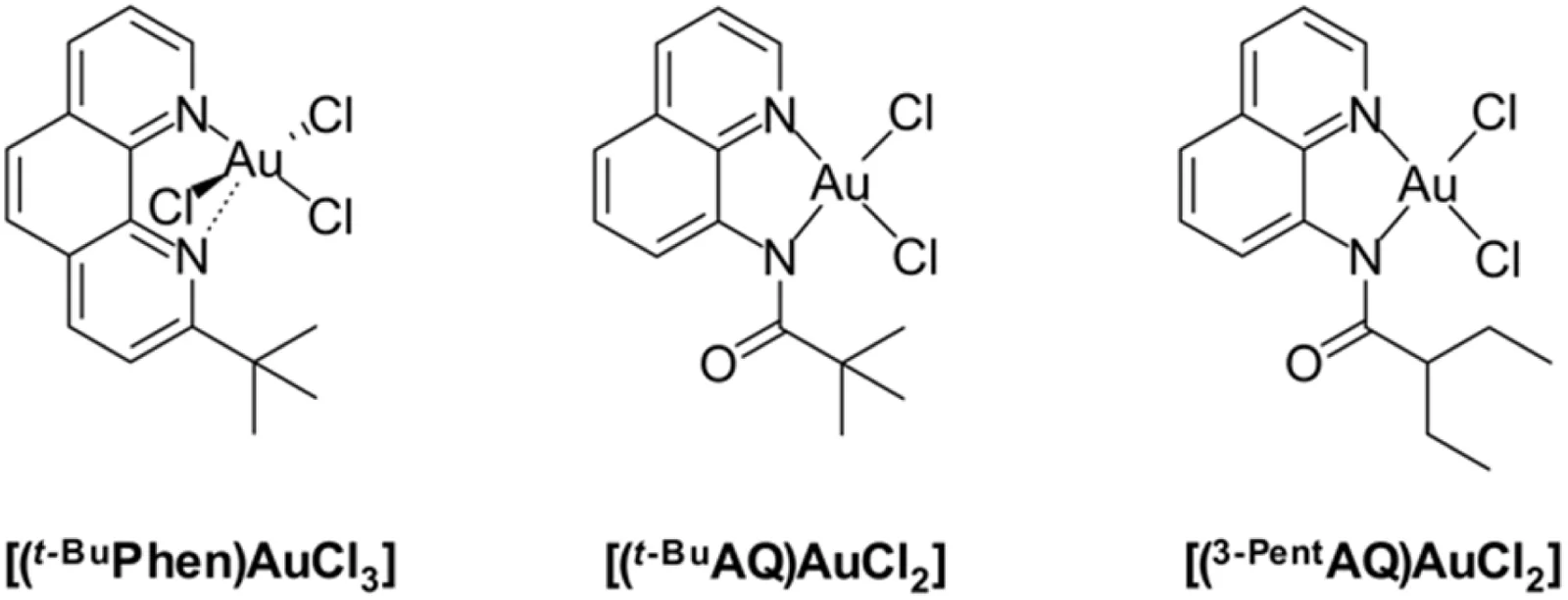
New gold(iii) complexes.

Amides of 8-aminoquinoline present a complementary sterically tunable bidentate *N*,*N*-donor system for gold(iii), and the resulting complexes have also shown potential as catalysts and anti-cancer agents.^13–17^ Gold(iii) complexes of 8-aminoquinoline and its amides, including sulfonamides, are square planar and cytotoxic (Fig. 3).^13–20^ While 8-aminoquinoline itself and its sulfonamides bind in a bidentate fashion, other reported amide derivatives act as tridentate ligands by incorporating an additional amine, pyridine, or phenyl donor alongside the two nitrogens of the 8-aminoquinoline core.^13–20^ We now report the synthesis of gold(iii) complexes from two new bidentate amides of 8-aminoquinoline bearing bulky substituents adjacent to the amide: 2,2-dimethyl-*N*-8-quinolinylpropanamide (^*t*-Bu^AQ-H) and 2-ethyl-*N*-8-quinolinylbutanamide (^3-Pent^AQ-H), affording [(^*t*-Bu^AQ)AuCl_2_] and [(^3-Pent^AQ)AuCl_2_] (Fig. 2).

**Fig. 3:**
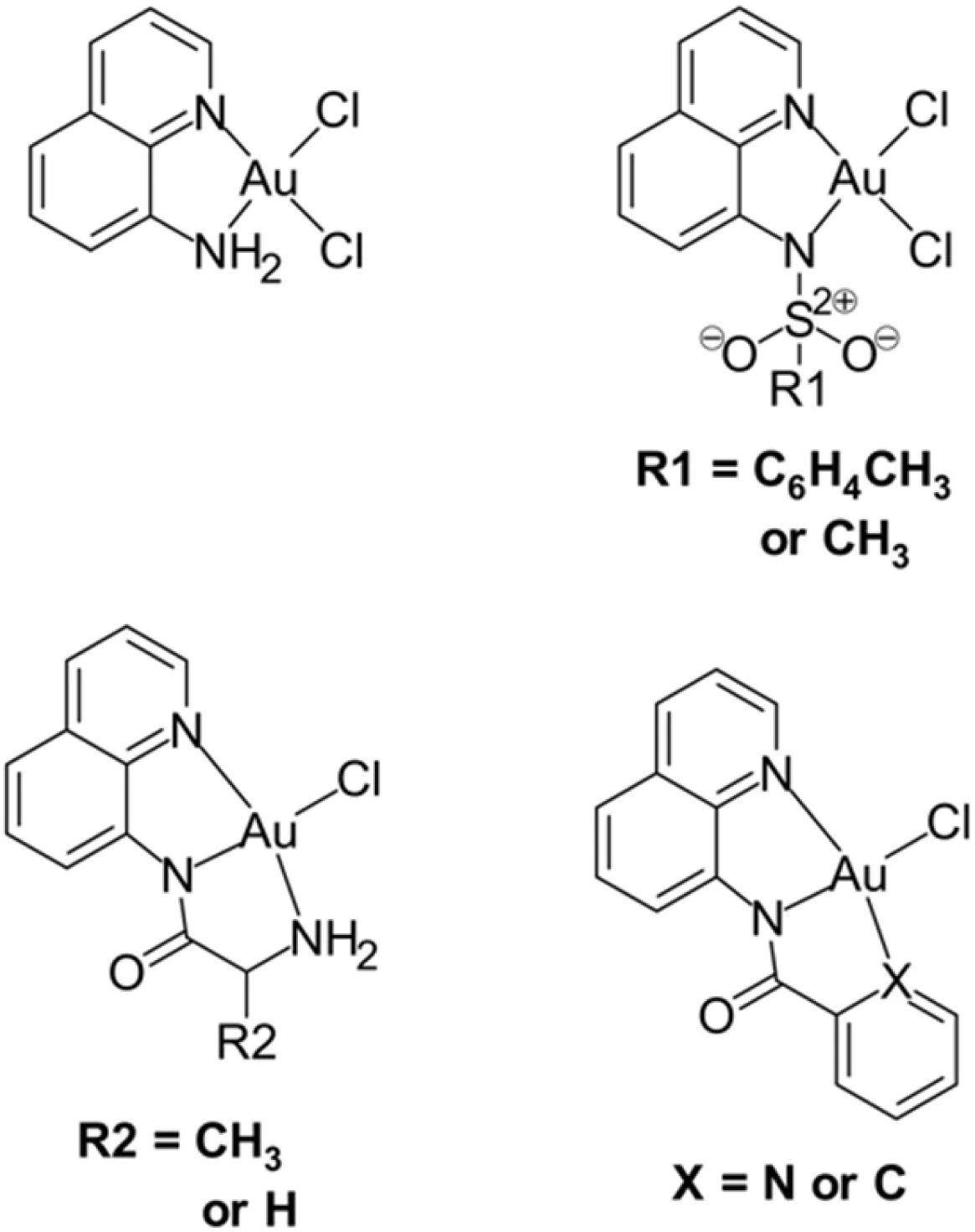
Gold(iii) complexes synthesized previously with ligands based on 8-aminoquinoline.^13–20^

## Results and discussion

The three gold(iii) complexes depicted in Fig. 2 ([(^*t*-Bu^Phen)AuCl_3_], [(^*t*-Bu^AQ)AuCl_2_], and [(^3-Pent^AQ)AuCl_2_]) were each synthesized by dissolving the appropriate ligand (^*t*-Bu^Phen, ^*t*-Bu^AQ, or ^3-Pent^AQ) in a minimal amount of an organic solvent and then mixing it with a concentrated aqueous solution of tetrachloroaurate ([AuCl_4_]^−^) from either KAuCl_4_ or NaAuCl_4_. It is important to minimize the amounts of the solvents to accommodate the precipitation of the product as the reaction proceeds. This dependence on product precipitation also causes the choice of organic solvent to be ligand dependent. Interestingly, higher yields of [(^*t*-Bu^Phen)AuCl_3_] and [(^*t*-Bu^AQ)AuCl_2_] were achieved by including NaHCO_3_ in the aqueous solution of [AuCl_4_]^−^, but the inclusion of NaHCO_3_ did not improve the yield of [(^3-Pent^AQ)AuCl_2_]. Therefore, the utility of adding NaHCO_3_ does not seem to depend on whether or not ligation requires deprotonation because only the quinolinyl amides (^R^AQ-H) require deprotonation.

Since the formation of [(^*t*-Bu^Phen)AuCl_3_] should not depend on deprotonation, the benefit of adding NaHCO_3_ likely relates either to solubility or to the speciation of [AuCl_4_]^−^ in solution.^21,22^ Previously reported ligations of phenanthroline ligands have not utilized NaHCO_3_.^7–10,12,23^ While ligation with just KAuCl_4_, NaAuCl_4_, or HAuCl_4_ have been reported, some other ligations of phenanthrolines have required AgBF_4_ to abstract a chloride from [AuCl_4_]^−^.^7–10,12^ Another phenanthroline ligation utilized HAuCl_4_ in an aqueous acetic acid solution with ceric ammonium nitrate.^23^ Presumably, all of these options affect the speciation of [AuCl_4_]^−^ while also impacting solubilities.^21,22^ After attempting multiple ligation strategies, including the use of HAuCl_4_ and a reaction with AgBF_4_, the best yields of [(^*t*-Bu^Phen)AuCl_3_] were achieved by using the procedure described above, starting with an aqueous solution of KAuCl_4_ and NaHCO_3_.

The [(^*t*-Bu^Phen)AuCl_3_] complex is the first example of gold(iii) binding to a phenanthroline with a *t*-butyl substituent or even a group with a quaternary carbon directly attached to either the 2- or 9- positions. As indicated by the drawing in Fig. 2 and as seen in the crystal structure (Fig. 4), the less sterically hindered phenanthroline nitrogen along with the three chlorides approximately form a square plane at the gold(iii) center. A further interaction could be considered between gold(iii) and the second phenanthroline nitrogen in an apical position because they are closer (2.786(3) Å) than the sum of their van der Waals radii (3.21 Å).^9^ Similar gold(iii) complexes of 2-substituted or 2,9-disubstituted phenanthrolines are considered to be pseudo five coordinate, and they contain only slightly shorter apical Au–N distances, ranging from 2.556(3) Å to 2.735(3) Å.^7–11^ As shown in Table 1, the gold(iii) complexes with 2-subsitituted phenanthroline ligands generally increase in Au–N_apical_ distance with an increase in the steric bulk of the substituent.^8,9,24^

**Fig. 4:**
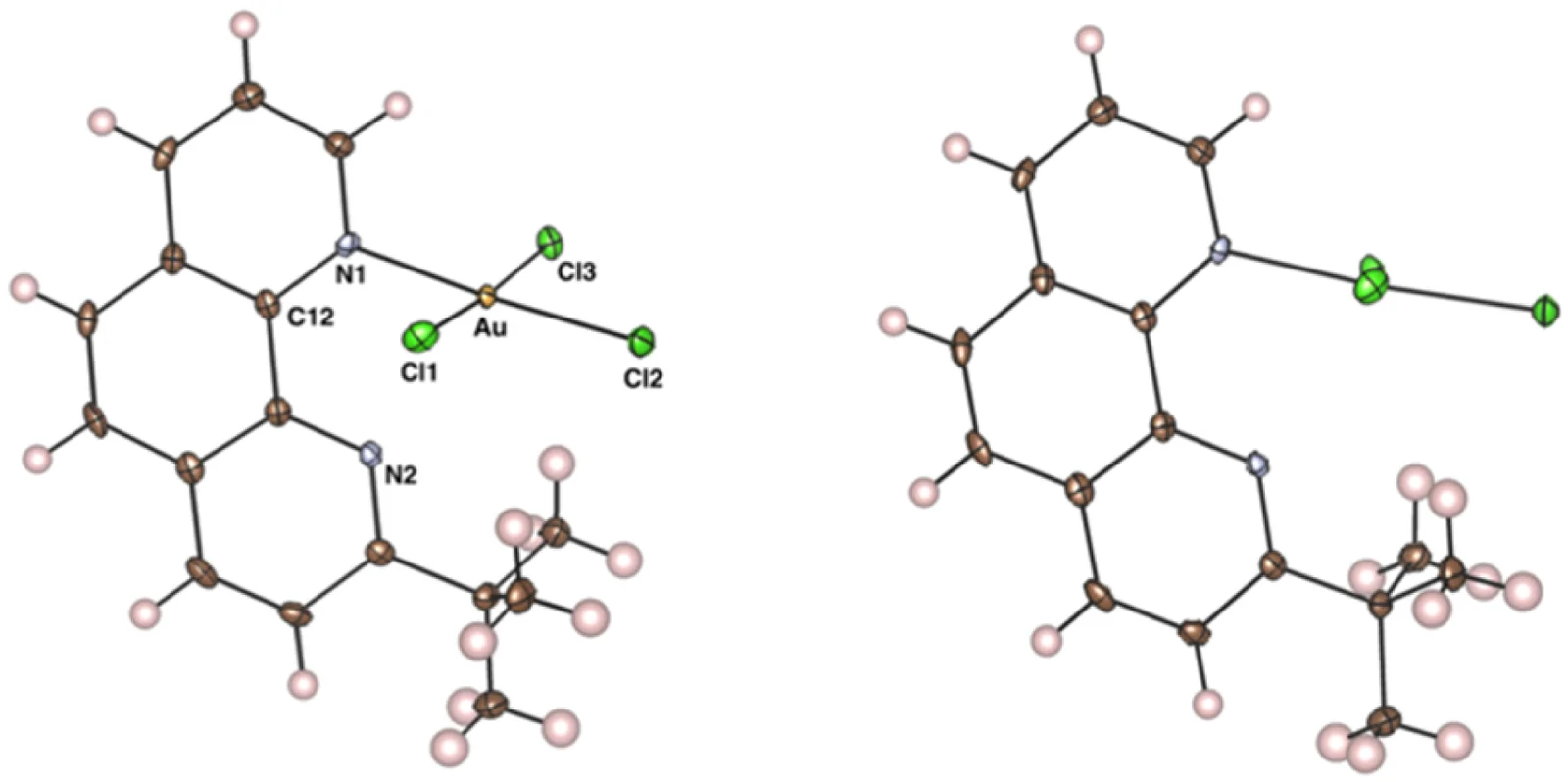
Two views of the single crystal X-ray diffraction structure of [(^*t*-Bu^Phen)AuCl_3_]. The view on the right is aligned with the Cl1–Au bond. The thermal ellipsoids are drawn at the 50% level. Selected bond lengths (Å) and angles (°): Au–N2 = 2.786(3), Au–N1 = 2.050(3), Au–Cl1 = 2.2832(10), Au–Cl2 = 2.2649(10), Au–Cl3 = 2.2918(9), N1–Au–Cl2 = 175.40(7), Cl1–Au–Cl3 = 175.55(3), N1–Au–Cl1 = 88.78(9), N1–Au–Cl3 = 89.08(8), Cl1–Au–Cl2 = 90.47(4), Cl2–Au–Cl3 = 91.35(4), N1–Au–N2 = 70.05(9), Cl1–Au–N1–C12 = 73.9(3). This results in a longer apical Au–N bond than in the other gold(iii)-phenanthroline complexes.^8,9^

**Table 1:** A comparison of Au–N_apical_ distances, *τ*_*δ*_, *τ*_5_, and square planar angles for 2-substituted phenanthroline complexes of AuCl_3_.^8,9,25,26^

2-Substituted phenanthroline complexes of AuCl_3_	Au–N_apical_ distance (Å)	*τ*_δ_	*τ*_5_	Angles between *trans* donors in square plane (°)	Angles between *cis* donors in square plane (°)
[(^*n*-Bu^Phen)AuCl_3_]	2.642	0.0559	0.0733	178.16, 173.76	87.2–90.8
[(^*sec*-Bu^Phen)AuCl_3_]	2.671	0.0599	0.0650	177.63, 173.73	88.9–90.7
[(^Ph^Phen)AuCl_3_]	2.735	0.0623	0.0228	176.26, 174.89	89.4–90.4
[(^*t*-Bu^Phen)AuCl_3_]	2.786	0.0641	0.00233	175.41, 175.55	88.8–91.4

To assess the effect of the apical nitrogen donor on the geometry at the gold(iii) center, it is useful to compare the *τ*_*δ*_ and *τ*_5_ indices for [(^*t*-Bu^Phen)AuCl_3_] with those of the other gold(iii) complexes of 2-substituted phenanthrolines as shown in Table 1.^8,9,25,26^ While by definition, neither of these indices can distinguish between square planar and square pyramidal, all of the *τ*_*δ*_ and *τ*_5_ values for these complexes are quite small (<0.1), indicating very little distortion from square planar or square pyramidal.^25,26^ Interestingly, there is a small increase in *τ*_*δ*_ and a bigger but still small decrease in *τ*_5_ with increasing steric bulk of the 2-substituted phenanthroline and increasing Au–N_apical_ distance.^8,9,24–26^ The decrease in *τ*_5_ likely indicates that increased steric bulk weakens the Au–N_apical_ interaction, and any Au–N_apical_ interaction in [(^*t*-Bu^Phen)AuCl_3_] must be very weak, since its *τ*_5_ is substantially lower than the others and approaches zero. The small increase in *τ*_*δ*_ with increasing steric bulk could reflect more general steric interactions between the phenanthroline substituent and the square plane.^24^

Within the square plane of [(^*t*-Bu^Phen)AuCl_3_], the Au–N bond length (2.050(3) Å) is similar to those of other gold(iii) complexes containing 2-substituted phenanthrolines (2.054(2)–2.060(3) Å); however, these are all shorter than the corresponding Au–N bond lengths in complexes of 2,9-disubstituted phenanthrolines (2.064(12)–2.09(1) Å).^8,9^ In the case of the 2,9-disubstituted phenanthrolines, greater steric bulk near the gold(iii) center likely leads to the lengthening of the basal Au–N bond because these ligands would otherwise be considered better donors based on electronics.

In the synthesis of [(^*t*-Bu^AQ)AuCl_2_] and [(^3-Pent^AQ)AuCl_2_] (Fig. 2), the amide undergoes deprotonation to form an amido donor, regardless of whether or not NaHCO_3_ is utilized as a base in the reaction mixture. In both of these complexes, the amides of 8-aminoquinoline bind in a bidentate manner; however, the different alkyl groups of the amides lead to dissimilar geometries and bonding within the ligand. In the case of [(^3-Pent^AQ)AuCl_2_], the amido is fairly close to planar, as seen in Fig. 5, with angles summing to 354.5°. The amido in [(^*t*-Bu^AQ)AuCl_2_] is less planar (Fig. 6) with angles summing to 348.6°, but this is still closer to planar (360°) than to pyramidal (328.5°). The greater planarity of the [(^3-Pent^AQ)AuCl_2_] amido indicates greater stabilization of the nitrogen lone pair through π-bonding. The filled π-orbitals of gold(iii) should minimize the amount of π-bonding with the amido lone pair.^27^ Furthermore, the similarities of the gold(iii) centers implies that the difference in amido planarity instead stems from differences between the ^3-Pent^AQ and ^*t*-Bu^AQ ligands. While the gold(iii)-amido bond is shorter (2.011(4) Å) in [(^3-Pent^AQ)AuCl_2_] as compared to 2.028(6) Å in [(^*t*-Bu^AQ)AuCl_2_], the shorter bond in [(^3-Pent^AQ)AuCl_2_] could be explained by a decrease in steric bulk proximal to the gold(iii) center and/or by a decrease in nitrogen-gold lone pair repulsions.

**Fig. 5:**
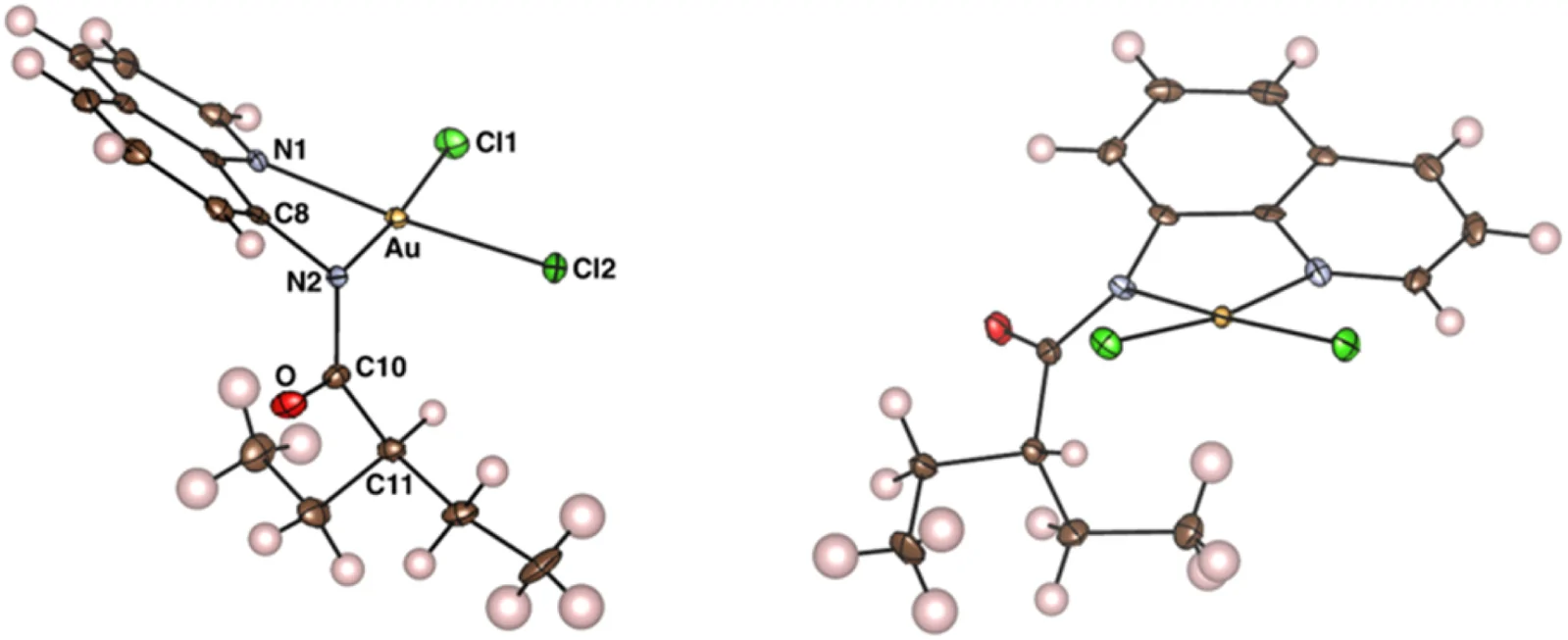
Two views of the single crystal X-ray diffraction structure of [(^3-Pent^AQ)AuCl_2_]. The thermal ellipsoids are drawn at the 50% level. Selected bond lengths (Å) and angles (°): Au–N1 = 2.027(4), Au–N2 = 2.011(4), Au–Cl1 = 2.2850(12), Au–Cl2 = 2.2712(12), N2–C8 = 1.424(6), N2–C10 = 1.392(6), C10–O = 1.230(6), N1–Au–Cl2 = 171.55(12), N2–Au–Cl1 = 175.04(11), N1–Au–N2 = 80.97(16), N1–Au–Cl1 = 94.09(12), N2–Au–Cl2 = 95.50(12), Cl1–Au–Cl2 = 89.32(5), C8–N2–Au = 108.9(3), C8–N2–C10 = 118.9(7), Au–N2–C10 = 126.7(3), N2–C10–C11 = 119.1(4), N2–C10–O = 120.3(4), O–C10–C11 = 120.6(4), Au–N2–C10–C11 = 4.7(6), Au–N2–C10–O = 172.8(4), C8–N2–C10–C11 = 155.6(4), C8–N2–C10–O = 21.9(7).

**Fig. 6:**
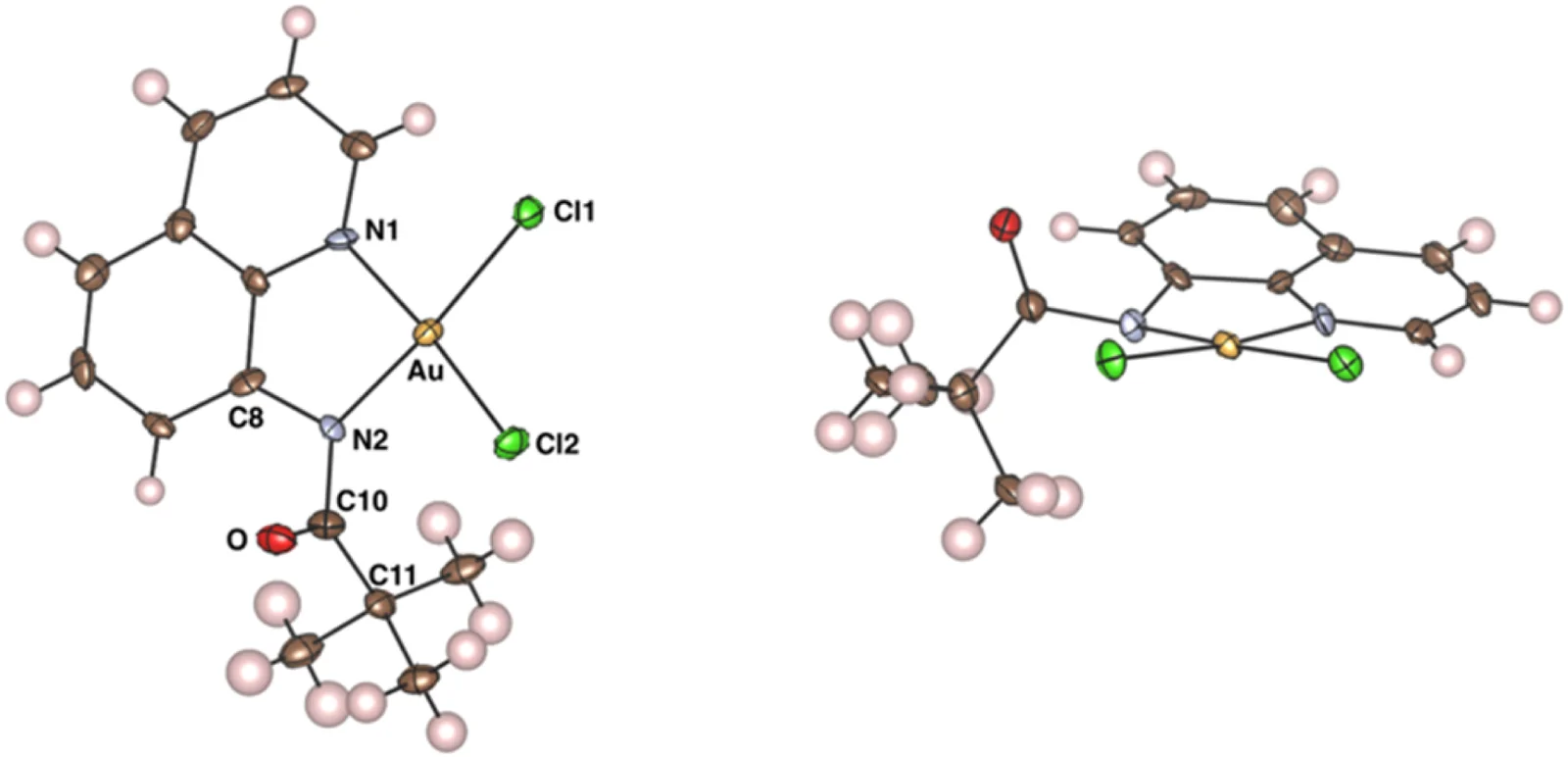
Two views of the single crystal X-ray diffraction structure of [(^*t*-Bu^AQ)AuCl_2_]. The thermal ellipsoids are drawn at the 50% level. Selected bond lengths (Å) and angles (°):Au–N1 = 2.033(7), Au–N2 = 2.028(6), Au–Cl1 = 2.309(3), Au–Cl2 = 2.281(3), N2–C8 = 1.392(10), N2–C10 = 1.460(10), C10–O = 1.207(11), N1–Au–Cl2 = 174.3(2), N2–Au–Cl1 = 174.79(18), N1–Au–N2 = 81.3(3), N1–Au–Cl1 = 93.55(19), N2–Au–Cl2 = 95.19(19), Cl1–Au–Cl2 = 89.96(7), C8–N2–Au = 110.5(5), C8–N2–C10 = 118.7(7), Au–N2–C10 = 119.4(5), N2–C10–C11 = 115.2(7), N2–C10–O = 120.2(8), O–C10–C11 = 123.9(8), Au–N2–C10–C11 = 99.3(9), Au–N2–C10–O = 84.9(10), C8–N2–C10–C11 = 120.6(9), C8–N2–C10–O = 55.2(13).

In further comparing the single crystal X-ray diffraction structures of [(^3-Pent^AQ)AuCl_2_] (Fig. 5) and of [(^*t*-Bu^AQ)AuCl_2_] (Fig. 6), the lone pair of the amido nitrogen does seem to be stabilized by greater π-bonding between the amido nitrogen (N2) and the carbonyl carbon (C10) within the ^3-Pent^AQ ligand as compared to within the ^*t*-Bu^AQ ligand. This is apparent from the shorter N2–C10 bond of 1.392(6) Å in [(^3-Pent^AQ)AuCl_2_] relative to 1.460(10) Å in [(^*t*-Bu^AQ)AuCl_2_]. As π-bonding increases between the amido and the carbonyl carbon in [(^3-Pent^AQ)AuCl_2_], this forces a decrease in π-bonding within the carbonyl causing the bond to be both longer and weaker than in [(^*t*-Bu^AQ)AuCl_2_]. The C10–O bond of the carbonyl in [(^3-Pent^AQ)AuCl_2_] is 1.230(6) Å as compared to 1.207(11) Å in [(^*t*-Bu^AQ)AuCl_2_]. The weakened carbonyl in [(^3-Pent^AQ)AuCl_2_] is also evident from its decreased infrared stretching frequency of 1640 cm^−1^ relative to 1700 cm^−1^ in [(^*t-*Bu^AQ)AuCl_2_].

In [(^3-Pent^AQ)AuCl_2_], π-donation from the amido lone pair to the carbonyl carbon is accommodated by the near coplanarity of the trigonal planes surrounding these atoms. The degree of coplanarity can be assessed by looking at the dihedral angles in the crystal structure (Fig. 5) that have the amido nitrogen and carbonyl carbon at their center. Perfect coplanarity would be indicated by angles of 0° and 180°. The Au–N2–C10–C11 angle of 4.7(6)° and the Au–N2–C10–O angle of 172.8(4)° come very close, while there is more deviation in the C8–N2–C10–O angle of 21.9(7)° and the C8–N2–C10–C11 angle of 155.6(4)°. In [(^*t*-Bu^AQ)AuCl_2_], steric repulsions between the *t*-butyl group and a chloride ligand seemingly force the atoms surrounding the carbonyl carbon and the amido nitrogen to be distinctly not coplanar as indicated in Fig. 6 by the Au–N2–C10–C11 dihedral angle of 99.3(9)°, the Au–N2–C10–O angle of 84.9(10)°, the C8–N2–C10–O angle of 120.6(9)°, and the C8–N2–C10–C11 angle of 55.2(13)°.

## Experimental

### General considerations

All manipulations were performed at room temperature and pressure unless otherwise noted. Previously published procedures were used to synthesize 2-*tert*-butyl-1,10-phenanthroline (^*t*-Bu^Phen)^28^ and 2,2-dimethyl-*N*-8-quinolinylpropanamide (^*t*-Bu^AQ-H).^29^ The procedure used to synthesize ^*t*-Bu^AQ-H was also applied to 2-ethyl-*N*-8-quinolinylbutanamide (^3-Pent^AQ-H), and the characterization of ^3-Pent^AQ-H matched the literature.^29^

Potassium tetrachloroaurate(iii) hydrate (99.99%-Au) (51%-Au) PURATREM was purchased from Strem Chemicals, while 99.9% sodium tetrachloroaurate(iii) dihydrate was acquired from Surepure Chemicals, LLC. Sodium bicarbonate (‘Baker Analyzed’ Reagent) was obtained from J.T. Baker Chemical. THF (Spectrophotometric Grade, 99.7+%, unstab.) was purchased from Alfa Aesar. Hexanes (AR (ACS)) were obtained from Macron Fine Chemicals. Acetone (Suitable for General Laboratory Use) and dichloromethane (BDH Chemicals Suitable for General Laboratory Use) were purchased from VWR. Acetonitrile (general laboratory grade, 99.5%) was acquired from BDH. NMR samples were prepared using ∼10 mg of sample dissolved in ∼0.8 mL of Thermo Scientific 99.9%-D acetone for NMR containing 0.03% TMS. The ^1^H NMR spectrum for [(^*t*-Bu^Phen)AuCl_3_] was obtained on a JEOL Eclipse 300 NMR Spectrometer with a 300 MHz Oxford magnet. All other NMR spectra were recorded using a Bruker Avance Neo NMR Spectrometer 400 NanoBay Console with a Fourier 400/54 Magnet. Volumetric glassware and serial dilutions were used to prepare samples for UV-vis spectra. These spectra were obtained using quartz cuvettes and a Thermo Electron Corporation Heλios γ spectrophotometer. IR samples were prepared by placing a concentrated CH_2_Cl_2_ solution of the compound on a NaCl plate and then evaporating the CH_2_Cl_2_. This process was repeated until the resulting film contained enough compound to obtain a spectrum. The IR spectra were obtained on a Thermo Nicolet Nexus 470 FT-IR E.S.P. To prepare the single-crystal X-ray diffraction samples, any solvent was decanted or removed by pipet, and the crystals were then coated with Paratone N oil. Elemental analyses were performed by Midwest Microlab.

#### Synthesis of [(^*t*-Bu^Phen)AuCl_3_]

To a 20 mL scintillation vial charged with a Teflon-coated stir bar was added 0.100 g (∼0.26 mmol) KAuCl_4_·*X* H_2_O, 0.0209 g (0.249 mmol) NaHCO_3_, and 5.0 mL of water. In a separate 20 mL glass vial, 0.0578 g (0.245 mmol) ^*t*-Bu^Phen were dissolved in 2.4 mL THF. The THF solution was added dropwise to the stirring aqueous solution, rinsing in with a minimal amount of additional THF. The vial was then wrapped in aluminum foil and allowed to stir for 4–5 days. Excess water was then added to precipitate the complex. The solid was isolated *via* vacuum filtration and rinsed twice each with water and hexanes. The solid was then dissolved in acetone to collect it from the filter paper, and the volatiles were subsequently removed under reduced pressure. This resulted in a yellow solid (0.052 g, 0.096 mmol, ∼39% yield). ^1^H NMR (300 MHz, acetone-*d*_6_): *δ* 9.84 (d, ^3^*J* = 6 Hz, 1H, *H*_Ar_), 9.11 (d, ^3^*J* = 8 Hz, 1H, *H*_Ar_), 8.68 (d, ^3^*J* = 9 Hz, 1H, *H*_Ar_), 8.25 (m, 4H, *H*_Ar_), 1.77 (s, 9H, C(C*H*_3_)_3_). ^13^C{^1^H} NMR (100 MHz, acetone-*d*_6_): *δ* 172.1, 153.4, 144.3, 141.3, 139.1, 134.0, 130.6, 129.9, 126.7, 126.6, 123.3, 40.0, 31.5. Anal. Calcd. for C_16_H_16_AuCl_3_N_2_: C, 35.61; H, 2.99; N, 5.19; Cl, 19.71. Found: C, 35.81; H, 3.04; N, 5.07; Cl, 19.41.

#### Synthesis of [(^*t*-Bu^AQ)AuCl_2_]

An aqueous solution of NaAuCl_4_·2 H_2_O (0.1040 g, 0.2615 mmol) and NaHCO_3_ (0.02730 g, 0.3250 mmol) was created using a minimal amount of water in a 20 mL vial. In a second vial, ^*t*-Bu^AQ-H (0.05315 g, 0.2328 mmol) was dissolved in a minimal amount of CH_3_OH. While stirring the first vial, the solution of ^*t*-Bu^AQ-H was added dropwise. The resulting solution was clear yellow. The reaction vial was then covered in aluminum foil and allowed to stir for 72 hours. During this time, the solution became green with a green precipitate. The precipitate was isolated *via* filtration and rinsed with hexanes. Subsequently, the green solid was dissolved in acetone and filtered. The volatiles in the filtrate were removed under reduced pressure. The resulting solid was redissolved in a minimal amount of THF and filtered to remove insoluble impurities. Hexanes were added to the filtrate until a dark green precipitate began to form. The vial was placed in a −2 °C freezer for 30 minutes to form additional precipitate. The liquid was decanted, and the solid was rinsed with hexanes. The volatiles were once again removed under reduced pressure, affording the product as a verdant green solid (0.0264 g, 0.0533 mmol, 23%). The product slowly decomposes at room temperature and was thus stored at −2 °C. Crystals for X-ray crystallography were generated *via* vapor diffusion of hexanes into a concentrated solution of [(^*t*-Bu^AQ)AuCl_2_] in CH_2_Cl_2_ at 7 °C. ^1^H NMR (400 MHz, acetone-*d*_6_): *δ* 9.39 (d, ^3^*J* = 6 Hz, 1H, *H*_Ar_), 8.89 (d, ^3^*J* = 9 Hz, 1H, *H*_Ar_), 8.05 (m, 1H, *H*_Ar_), 7.59 (*t*_app_, ^3^*J* = 8 Hz, 1H, *H*_Ar_), 7.49 (d, ^3^*J* = 8 Hz, 1H, *H*_Ar_), 7.12 (d, ^3^*J* = 8 Hz, 1H, *H*_Ar_), 1.39 (s, 9H, C(C*H*_3_)_3_). ^13^C{^1^H} NMR (100 MHz, acetone-*d*_6_): *δ* 192.5, 151.6, 147.8, 144.5, 133.0, 131.6, 124.0, 119.9, 115.4, 44.7, 28.4. ^1^H–^13^C HSQC NMR (400/100 MHz, acetone-*d*_6_) *δ*_C_/*δ*_H_ 147.8/9.39 (+, CH), 144.5/8.89 (+, CH), 131.6/7.59 (+, CH), 124.0/8.05 (+, CH), 119.9/7.49 (+, CH), 115.4/7.12 (+, CH), 28.4/1.39 (+, CH_3_). UV/vis (0.1 mM CH_2_Cl_2_) *λ*_max_ in nm (log *ε*): 321.5 (3.6), 441.0 (3.1), 711.0 (2.4). IR(NaCl): *ν*_CO_ = 1700 cm^−1^. Anal. Calcd. for C_14_H_15_AuCl_2_N_2_O: C, 33.96; H, 3.05; N, 5.66. Found: C, 34.28; H, 3.07; N, 5.62.

#### Synthesis of [(^3-Pent^AQ)AuCl_2_]

In a 20 mL scintillation vial, ^3-Pent^AQ-H (0.1027 g, 0.4238 mmol) was dissolved in a minimal amount of CH_3_CN, generating a light yellow solution. In a separate 20 mL vial, NaAuCl_4_·2 H_2_O (0.1815 g, 0.4563 mmol) and NaHCO_3_ (0.0397 g, 0.473 mmol) were dissolved in a minimal amount of distilled water. This solution was dark orange. The organic solution was added dropwise to the stirring aqueous solution. The vial containing the resulting solution was covered with aluminum foil, and it was allowed to stir for 170 hours. At the end of this time, the solution was dark yellow with an olive green precipitate. The solution was removed *via* filtration through a glass microfiber filter to isolate the olive green solid. This solid was then dissolved in acetone and filtered through the microfiber filter into a new flask. All volatiles were removed, and an olive green solid was obtained (0.140 g, 0.275 mmol, 64.9%). Crystals for X-ray crystallography were obtained *via* vapor diffusion of hexanes into a concentrated CH_2_Cl_2_ solution of [(^3-Pent^AQ)AuCl_2_]_._^1^H NMR (400 MHz, acetone-*d*_6_): *δ* 9.46 (d, ^3^*J* = 6 Hz, 1H, *H*_Ar_), 8.97 (d, ^3^*J* = 8 Hz, 1H, *H*_Ar_), 8.47 (dd, ^3^*J* = 8 Hz, ^4^*J* = 1 Hz, 1H, *H*_Ar_), 8.07 (m, 1H, *H*_Ar_), 7.76 (m, 2H, *H*_Ar_), 3.52 (m, 1H, C*H*(CH_2_CH_3_)_2_), 1.63 (m, 4H, CH(C*H*_2_CH_3_)_2_), 0.87 (t, 6H, CH(CH_2_C*H*_3_)_2_). ^13^C{^1^H} NMR (100 MHz, acetone-*d*_6_): *δ* 181.7, 148.9, 148.8, 144.8, 131.8, 131.0, 125.1, 123.8, 123.7, 51.8, 27.0, 12.2. ^1^H–^13^C HSQC NMR (400/100 MHz, acetone-*d*_6_) *δ*_C_/*δ*_H_ 148.8/9.46 (+, CH), 144.8/8.97 (+, CH), 131.0/7.76 (+, CH), 125.1/8.47 (+, CH), 123.8/8.07 (+, CH), 123.7/7.76 (+, CH), 51.8/3.52 (+, CH), 27.0/1.63(−, CH_2_), 12.2/0.87 (+, CH_3_). UV/vis (0.1 mM CH_2_Cl_2_) *λ*_max_ in nm (log *ε*): 319.0 (3.5), 384.0 (3.4). IR(NaCl): *ν*_CO_ = 1640 cm^−1^. Anal. Calcd. for C_15_H_17_AuCl_2_N_2_O: C, 35.38; H, 3.37; N, 5.50. Found: C, 35.09; H, 3.38; N, 5.26.

## Conclusions

The gold(iii) complexes [(^*t*-Bu^Phen)AuCl_3_], [(^*t*-Bu^AQ)AuCl_2_], and [(^3-Pent^AQ)AuCl_2_] were each synthesized by mixing an organic solution of the ligand with an aqueous solution of [AuCl_4_]^−^. The optimized syntheses of [(^*t*-Bu^Phen)AuCl_3_] and [(^*t*-Bu^AQ)AuCl_2_] included the addition of NaHCO_3_ to the aqueous solution, but this was not consistent with a simple base effect because only the syntheses of [(^*t*-Bu^AQ)AuCl_2_] and [(^3-Pent^AQ)AuCl_2_] required deprotonation of the ligand. Both [(^*t*-Bu^AQ)AuCl_2_] and [(^3-Pent^AQ)AuCl_2_] are square planar with the amide of 8-aminoquinoline binding in a bidentate manner. Gold(iii) complexes similar to [(^*t*-Bu^Phen)AuCl_3_] of 2-substituted phenanthrolines are generally considered pseudo five coordinate due to weak chelation by the more sterically hindered nitrogen of the phenanthroline, but this interaction is even weaker in [(^*t*-Bu^Phen)AuCl_3_].^8,9^ In general, analyses of [(^*t*-Bu^Phen)AuCl_3_], [(^*t*-Bu^AQ)AuCl_2_], and [(^3-Pent^AQ)AuCl_2_] indicated that the increased sterics associated with the presence of a *t*-butyl group near the gold(iii) center had a substantial impact on bond lengths, angles, and degree of bonding. This could be an important aspect to consider when choosing gold(iii) complexes to be tested as anti-cancer agents.^1–3,6^

## Author contributions

D. R. W. was involved in every aspect of the project except for obtaining the X-ray crystallographic data. A. L. R. obtained the X-ray crystallographic data but died before being able to review any drafts of this manuscript. The remaining authors contributed to the investigation. All authors except A. L. R. (deceased) read and agreed to the final manuscript.

## Conflicts of interest

The authors are unaware of any conflicts of interest.

## Supplementary Material

RA-016-D6RA07844C-s001

RA-016-D6RA07844C-s002

## Data Availability

CCDC 2237680, 2560842 and 2560843 contain the supplementary crystallographic data for this paper.^30–32^ NMR, IR, and UV-vis spectra as well as crystallographic data are included in the supplementary information (SI). Supplementary information is available. See DOI: https://doi.org/10.1039/d6ra07844c.

## References

[cit1] Moreno-Alcántar G., Picchetti P., Casini A. (2023). Angew. Chem., Int. Ed..

[cit2] Zhang J., Li Y., Fang R., Wei W., Wang Y., Jin J., Yang F., Chen J. (2022). Front. Pharmacol.

[cit3] Zou T., Lum C. T., Lok C.-N., Zhang J.-J., Che C.-M. (2015). Chem. Soc. Rev..

[cit4] Praveen C., Dupeux A., Michelet V. (2021). Chem.–Eur. J..

[cit5] Fricke C., Reid W. B., Schoenebeck F. (2020). Eur. J. Org. Chem..

[cit6] Da Silva Maia P. I., Deflon V. M., Abram U. (2014). Future Med. Chem..

[cit7] Hudson Z. D., Sanghvi C. D., Rhine M. A., Ng J. J., Bunge S. D., Hardcastle K. I., Saadein M. R., MacBeth C. E., Eichler J. F. (2009). Dalton Trans..

[cit8] Krause J. A., Zhao D., Chatterjee S., Falcon R., Stoltz K., Warren J. C., Wiswell S. E., Connick W. B., Collins S. N. (2014). Acta Crystallogr., Sect. C:Struct. Chem..

[cit9] Olsen P. M., Ruiz C., Lussier D., Le B. K., Angel N., Smith M., Hwang C. B., Khatib R., Jenkins J., Adams K., Getcher J., Tham F., Chen Z. G., Wilson E. H., Eichler J. F. (2014). J. Inorg. Biochem..

[cit10] Robinson W. T., Sinn E. (1975). J. Chem. Soc., Dalton Trans..

[cit11] Sanghvi C. D., Olsen P. M., Elix C., Peng S. B., Wang D., Chen Z. G., Shin D. M., Hardcastle K. I., MacBeth C. E., Eichler J. F. (2013). J. Inorg. Biochem..

[cit12] Abbate F., Orioli P., Bruni B., Marcon G., Messori L. (2000). Inorg. Chim. Acta.

[cit13] Yang T., Chao T., Zhang J., Lin L., Zhang X., Liu Q., Ding J., Xu Q., Guo Z. (2003). Dalton Trans..

[cit14] Casado-Sánchez A., Martín-Santos C., Padrón J. M., Mas-Ballesté R., Navarro-Ranninger C., Alemán J., Cabrera S. (2017). J. Inorg. Biochem..

[cit15] Kilpin K. J., Jarman B. P., Henderson W., Nicholson B. K. (2011). Appl. Organomet. Chem..

[cit16] Martín-Santos C., Michelucci E., Marzo T., Messori L., Szumlas P., Bednarski P. J., Mas-Ballesté R., Navarro-Ranninger C., Cabrera S., Alemán J. (2015). J. Inorg. Biochem..

[cit17] Niizeki R., Higashida K., Mejri E., Sawamura M., Shimizu Y. (2022). Synlett.

[cit18] Kilpin K. J., Henderson W., Nicholson B. K. (2008). Dalton Trans..

[cit19] Usón R., Vicente J., Chicote M. T. (1981). J. Organomet. Chem..

[cit20] Wasi N., Singh H. B., Gajanana A., Raichowdhary A. N. (1987). Inorg. Chim. Acta.

[cit21] Mironov I. V., Makotchenko E. V. (2009). J. Solution Chem..

[cit22] Robb W. (1967). Inorg. Chem..

[cit23] Dey D., Al-Hunaiti A., Gopal V., Perumalsamy B., Balakrishnan G., Ramasamy T., Dharumadurai D., Biswas B. (2020). J. Mol. Struct..

[cit24] Eliel E. L. and Wilen S. H., Stereochemistry of Organic Compounds, John Wiley & Sons, Inc., United States of America, 1994

[cit25] Addison A. W., Nageswara Rao T., Reedijk J., van Rijn J., Verschoor G. C. (1984). J. Chem. Soc., Dalton Trans..

[cit26] Reineke M. H., Sampson M. D., Rheingold A. L., Kubiak C. P. (2015). Inorg. Chem..

[cit27] Gray H. B. , Chemical Bonds: an Introduction to Atomic and Molecular Structure, University Science Books, 1994

[cit28] Gimeno L., Blart E., Rebilly J. N., Coupeau M., Allain M., Roisnel T., Quarré De Verneuil A., Gourlaouen C., Daniel C., Pellegrin Y. (2020). Chem.–Eur. J..

[cit29] Xiong H.-Y., Besset T., Cahard D., Pannecoucke X. (2015). J. Org. Chem.

[cit30] Rheingold A. L. and Weinberg D. R., CCDC 2237680: Experimental Crystal Structure Determination, 2023, 10.5517/ccdc.csd.cc2f3h79

[cit31] Rheingold A. L. and Weinberg D. R., CCDC 2560842: Experimental Crystal Structure Determination, 2026, 10.5517/ccdc.csd.cc2ryrt9

[cit32] Rheingold A. L. and Weinberg D. R., CCDC 2560843: Experimental Crystal Structure Determination, 2026, 10.5517/ccdc.csd.cc2ryrvb

