# Supplementary material for: Gold(iii) complexes of 2-tert-butyl-1,10-phenanthroline and of N-(8-quinolinyl)amides: syntheses and structures
Source: RSC Adv. 2026 Sep 7;16(43):53268–73. doi: 10.1039/d6ra07844c (PMC13549306; doi:10.1039/d6ra07844c)
Supplement: Supplementary file 1 [file RA-016-D6RA07844C-s001.pdf]

Supplementary Information (SI) for RSC Advances.
This journal is © The Royal Society of Chemistry 2026

Supplementary Information

Gold(III) complexes of 2-*tert*-butyl-1,10-phenanthroline and of *N*-(8-quinolinyl)amides: Syntheses and structures

David R. Weinberg,*[a] Kaitlyn M. Rutter,[a] Joshua E. Thompson,[a] Matt Sleck,[a] Nicholas A. Curry,[a] Alex E. Eckberg,[a] Julian A. Manila,[a] Hayden R. Murphy,[a] Karina Saucedo Chavez,[a] Siya R. Salunke,[a] Zoe Steele,[a] and Arnold L. Rheingold†[b]

[a] Colorado Mesa University, Department of Physical and Environmental Sciences, 1100 North Ave., Grand Junction, CO 81501, U.S.A.

[b] University of California, San Diego, Department of Chemistry and Biochemistry, 9500 Gilman Dr., La Jolla, CA 92093, U.S.A.

† *Deceased: March 3rd, 2024*

Table of Contents

I) [($^{t\text{-Bu}}$Phen)$AuCl_3$]……S2
- A) Single Crystal X-ray Diffraction……S2
- B) ^{1}H NMR Spectrum……S8
- C) ^{13}C{^{1}H} NMR Spectrum……S9

II) [($^{t\text{-Bu}}$AQ)$AuCl_2$]……S10
- A) Single Crystal X-ray Diffraction……S10
- B) ^{1}H NMR Spectrum……S17
- C) ^{13}C{^{1}H} NMR Spectrum……S18
- D) ^{1}H—^{13}C HSQC NMR Spectrum……S19
- E) UV-Vis Spectrum……S20
- F) IR Spectrum……S21

III) [($^{3\text{-Pent}}$AQ)$AuCl_2$]
- A) Single Crystal X-ray Diffraction……S22
- B) ^{1}H NMR Spectrum……S27
- C) ^{13}C{^{1}H} NMR Spectrum……S28
- D) ^{1}H—^{13}C HSQC NMR Spectrum……S29
- E) UV-Vis Spectrum……S30
- F) IR Spectrum……S31

I) [($^{t\text{-}Bu}$Phen)AuCl$_3$]

A) Single Crystal X-ray Diffraction

Table S1. Crystal data and structure refinement for [($^{t\text{-}Bu}$Phen)AuCl$_3$].

Identification code	comesa01	
Empirical formula	C16 H16 Au Cl3 N2	
Formula weight	539.62	
Temperature	100(2) K	
Wavelength	0.71073 Å	
Crystal system	Triclinic	
Space group	P1	
Unit cell dimensions	a = 8.5447(15) Å	α = 98.088(7)°
	b = 9.0033(15) Å	β = 103.667(8)°
	c = 11.935(2) Å	γ = 102.264(7)°
Volume	854.0(3) Å^3	
Z	2	
Density (calculated)	2.098 g/cm^3	
Absorption coefficient	9.079 mm^{-1}	
F(000)	512	
Crystal size	0.28 x 0.26 x 0.22 mm^3	
Crystal color, habit	Yellow block	
Theta range for data collection	2.36 to 26.47°	
Index ranges	-10<=h<=10, -11<=k<=7, -14<=l<=14	
Reflections collected	12622	
Independent reflections	3471 [R(int) = 0.0287]	
Completeness to theta = 25.00°	99.0 %	
Absorption correction	Multi-scan	
Max. and min. transmission	0.2400 and 0.1854	
Refinement method	Full-matrix least-squares on F^2	
Data / restraints / parameters	3471 / 0 / 202	
Goodness-of-fit on F^2	1.040	
Final R indices [I>2sigma(I)]	R1 = 0.0165, wR2 = 0.0394	
R indices (all data)	R1 = 0.0179, wR2 = 0.0399	
Largest diff. peak and hole	2.091 and -1.290 e Å^{-3}	

Table S2. Atomic coordinates (x 10^4) and equivalent isotropic displacement parameters ($Å^2$x 10^3) for [($^{t\text{-Bu}}$Phen)$AuCl_3$]. U(eq) is defined as one third of the trace of the orthogonalized U^{ij} tensor.

	x	y	z	U(eq)
Au(1)	1752(1)	1736(1)	1668(1)	9(1)
Cl(1)	942(1)	3416(1)	2895(1)	17(1)
Cl(2)	1125(1)	-155(1)	2676(1)	19(1)
Cl(3)	2395(1)	60(1)	320(1)	14(1)
N(1)	2120(3)	3406(3)	686(2)	9(1)
N(2)	4936(3)	3710(3)	2609(2)	10(1)
C(1)	899(4)	3181(4)	-299(2)	11(1)
C(2)	854(4)	4264(4)	-1018(3)	13(1)
C(3)	2091(4)	5610(4)	-692(3)	13(1)
C(4)	3403(4)	5857(3)	331(3)	11(1)
C(5)	4736(4)	7234(3)	666(3)	13(1)
C(6)	6011(4)	7456(3)	1635(3)	13(1)
C(7)	6089(4)	6298(3)	2330(3)	11(1)
C(8)	7448(4)	6455(4)	3308(3)	15(1)
C(9)	7540(4)	5266(4)	3886(3)	14(1)
C(10)	6264(4)	3871(3)	3502(2)	11(1)
C(11)	4827(4)	4890(3)	2028(2)	10(1)
C(12)	3422(4)	4703(3)	1022(2)	9(1)
C(13)	6339(4)	2499(4)	4119(3)	13(1)
C(14)	5944(4)	1008(4)	3193(3)	17(1)
C(15)	5048(4)	2412(4)	4831(3)	19(1)
C(16)	8049(4)	2671(4)	4960(3)	19(1)

Table S3. Bond lengths [Å] and angles [°] for [($^{t\text{-Bu}}$Phen)$AuCl_3$].

Au(1)-N(1)	2.049(2)
Au(1)-Cl(2)	2.2649(8)
Au(1)-Cl(1)	2.2832(8)
Au(1)-Cl(3)	2.2918(8)
N(1)-C(1)	1.332(4)
N(1)-C(12)	1.367(4)
N(2)-C(10)	1.327(4)
N(2)-C(11)	1.355(4)
C(1)-C(2)	1.388(4)
C(2)-C(3)	1.365(4)
C(3)-C(4)	1.403(4)
C(4)-C(12)	1.415(4)
C(4)-C(5)	1.428(4)
C(5)-C(6)	1.346(4)
C(6)-C(7)	1.424(4)
C(7)-C(8)	1.405(4)
C(7)-C(11)	1.416(4)
C(8)-C(9)	1.359(4)
C(9)-C(10)	1.413(4)
C(10)-C(13)	1.529(4)
C(11)-C(12)	1.444(4)
C(13)-C(16)	1.528(4)
C(13)-C(14)	1.533(4)
C(13)-C(15)	1.540(4)
N(1)-Au(1)-Cl(2)	175.41(7)
N(1)-Au(1)-Cl(1)	88.79(7)
Cl(2)-Au(1)-Cl(1)	90.47(3)
N(1)-Au(1)-Cl(3)	89.07(7)
Cl(2)-Au(1)-Cl(3)	91.36(3)
Cl(1)-Au(1)-Cl(3)	175.55(3)
C(1)-N(1)-C(12)	121.4(3)
C(1)-N(1)-Au(1)	113.9(2)
C(12)-N(1)-Au(1)	124.56(19)

C(10)-N(2)-C(11)	119.3(3)
N(1)-C(1)-C(2)	122.0(3)
C(3)-C(2)-C(1)	118.6(3)
C(2)-C(3)-C(4)	120.2(3)
C(3)-C(4)-C(12)	119.3(3)
C(3)-C(4)-C(5)	121.0(3)
C(12)-C(4)-C(5)	119.7(3)
C(6)-C(5)-C(4)	121.0(3)
C(5)-C(6)-C(7)	120.6(3)
C(8)-C(7)-C(11)	116.7(3)
C(8)-C(7)-C(6)	122.1(3)
C(11)-C(7)-C(6)	121.1(3)
C(9)-C(8)-C(7)	120.1(3)
C(8)-C(9)-C(10)	119.8(3)
N(2)-C(10)-C(9)	121.4(3)
N(2)-C(10)-C(13)	117.3(3)
C(9)-C(10)-C(13)	121.3(3)
N(2)-C(11)-C(7)	122.5(3)
N(2)-C(11)-C(12)	119.8(3)
C(7)-C(11)-C(12)	117.6(3)
N(1)-C(12)-C(4)	118.3(3)
N(1)-C(12)-C(11)	121.8(3)
C(4)-C(12)-C(11)	119.8(3)
C(16)-C(13)-C(10)	113.0(3)
C(16)-C(13)-C(14)	107.6(3)
C(10)-C(13)-C(14)	109.3(2)
C(16)-C(13)-C(15)	108.6(3)
C(10)-C(13)-C(15)	107.2(2)
C(14)-C(13)-C(15)	111.2(3)

Table S4. Anisotropic displacement parameters ($Å^2$x 10^3) for [($^{t\text{-Bu}}$Phen)$AuCl_3$]. The anisotropic displacement factor exponent takes the form: $-2\pi^2[h^2 a^{*2}U^{11} + ... + 2 h k a^* b^* U^{12}]$

	U^{11}	U^{22}	U^{33}	U^{23}	U^{13}	U^{12}
Au(1)	10(1)	9(1)	8(1)	2(1)	2(1)	0(1)
Cl(1)	22(1)	22(1)	12(1)	3(1)	7(1)	8(1)
Cl(2)	22(1)	16(1)	14(1)	6(1)	3(1)	-2(1)
Cl(3)	15(1)	10(1)	16(1)	2(1)	6(1)	2(1)
N(1)	12(1)	10(1)	6(1)	3(1)	4(1)	3(1)
N(2)	10(1)	10(1)	9(1)	2(1)	4(1)	1(1)
C(1)	11(2)	12(2)	10(1)	1(1)	4(1)	3(1)
C(2)	13(2)	16(2)	11(1)	2(1)	5(1)	6(1)
C(3)	17(2)	15(2)	14(2)	8(1)	10(1)	8(1)
C(4)	13(2)	10(1)	13(1)	3(1)	8(1)	5(1)
C(5)	17(2)	10(2)	19(2)	6(1)	12(1)	6(1)
C(6)	14(2)	6(1)	19(2)	-2(1)	9(1)	-1(1)
C(7)	14(2)	10(2)	12(1)	-1(1)	9(1)	4(1)
C(8)	13(2)	13(2)	14(2)	-4(1)	5(1)	-3(1)
C(9)	12(2)	19(2)	9(1)	0(1)	2(1)	0(1)
C(10)	12(2)	13(2)	8(1)	1(1)	5(1)	2(1)
C(11)	12(2)	11(1)	9(1)	1(1)	7(1)	4(1)
C(12)	12(1)	9(1)	10(1)	0(1)	7(1)	3(1)
C(13)	12(2)	15(2)	11(2)	4(1)	4(1)	2(1)
C(14)	19(2)	14(2)	15(2)	3(1)	3(1)	4(1)
C(15)	18(2)	22(2)	19(2)	9(1)	9(1)	4(1)
C(16)	17(2)	20(2)	18(2)	6(1)	1(1)	5(1)

Table S5. Hydrogen coordinates (x 10^4) and isotropic displacement parameters ($Å^2$ x 10^3) for [($^{t\text{-}Bu}$Phen)$AuCl_3$].

	x	y	z	U(eq)
H(1A)	32	2250	-515	14
H(2A)	-19	4074	-1724	15
H(3A)	2063	6380	-1159	15
H(5A)	4722	8005	198	16
H(6A)	6867	8395	1857	16
H(8A)	8304	7394	3566	18
H(9A)	8460	5372	4547	17
H(14A)	6770	1103	2743	25
H(14B)	4830	842	2659	25
H(14C)	5979	125	3585	25
H(15A)	5380	3340	5457	28
H(15B)	4989	1486	5180	28
H(15C)	3953	2353	4308	28
H(16A)	8894	2783	4526	28
H(16B)	8036	1748	5310	28
H(16C)	8315	3592	5585	28

I) [(${}^{t\text{-Bu}}$Phen)$AuCl_3$]

B) ${}^1H$ NMR Spectrum

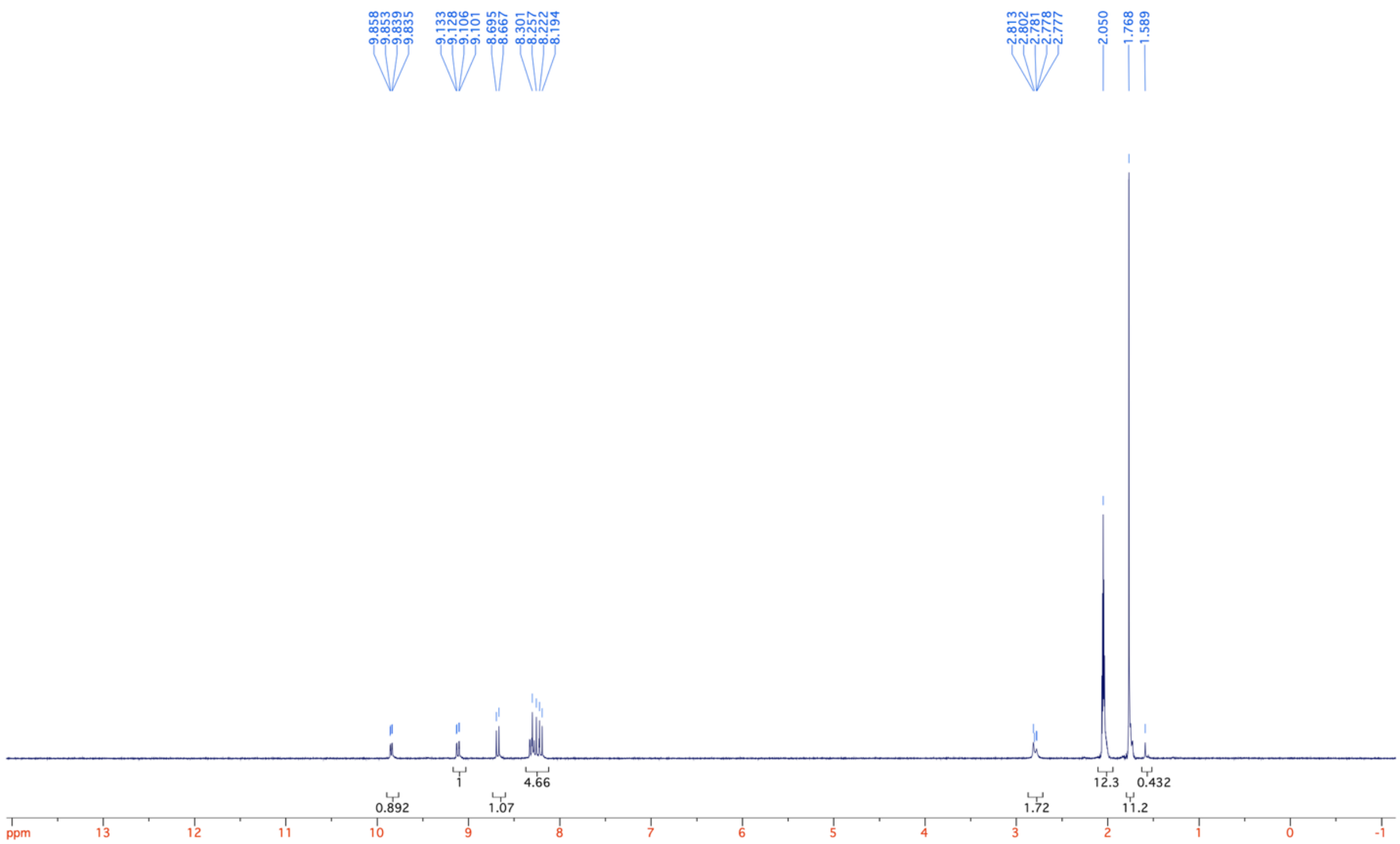

Figure S1. ${}^1H$ NMR spectrum of [(${}^{t\text{-Bu}}$Phen)$AuCl_3$] in acetone-d_6.

I) [($^{t\text{-Bu}}$Phen)AuCl$_3$]

C) $^{13}C\{^1H\}$ NMR Spectrum

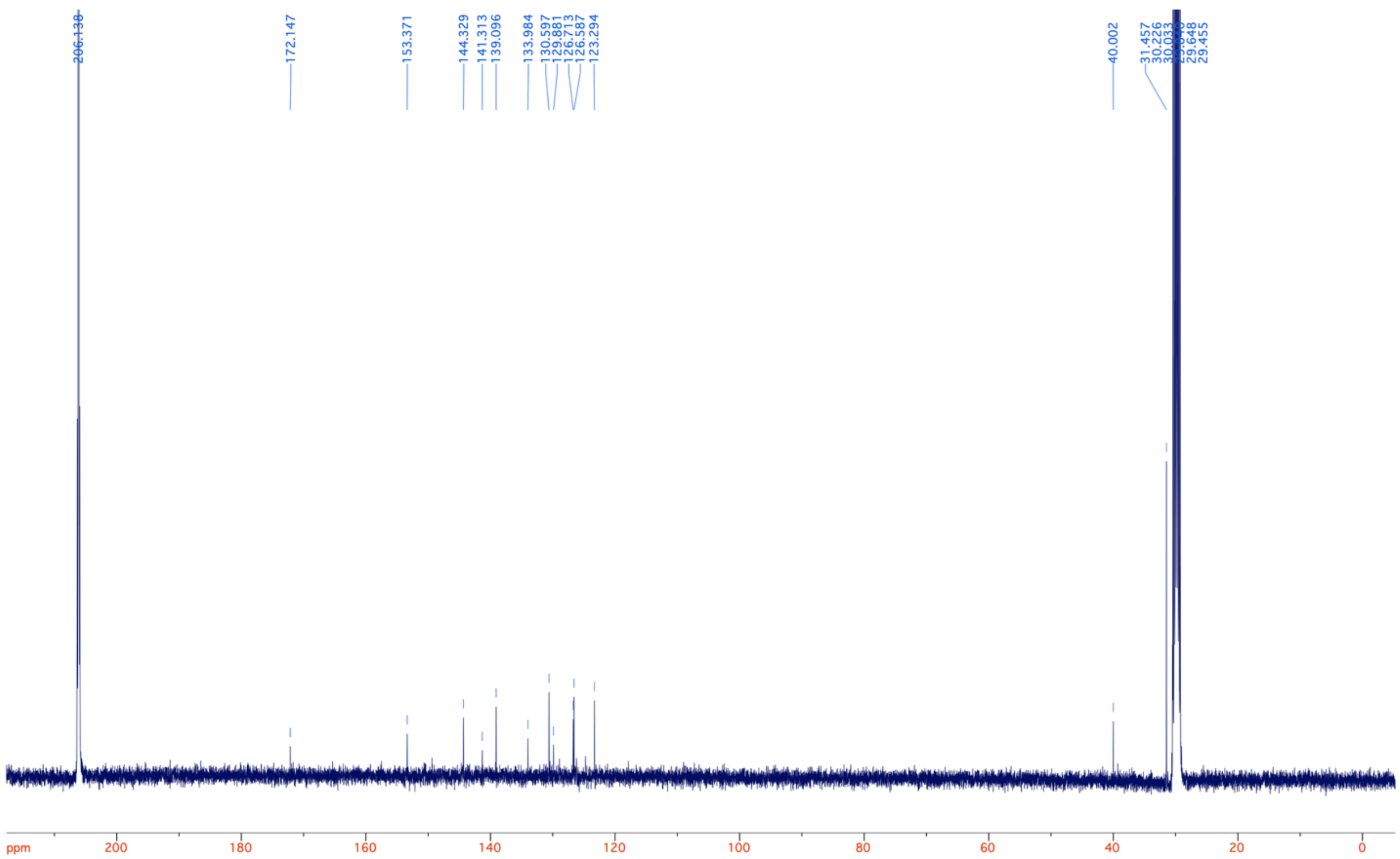

Figure S2. $^{13}C\{^1H\}$ NMR Spectrum of [($^{t\text{-Bu}}$Phen)AuCl$_3$] in acetone-d_6.

II) [($^{t\text{-Bu}}$AQ)$AuCl_2$]

A) Single Crystal X-ray Diffraction

Table S6. Crystal data and structure refinement for [($^{t\text{-Bu}}$AQ)$AuCl_2$].

Identification code	KM6 Comp2	
Empirical formula	C14 H15 Au Cl2 N2 O	
Formula weight	495.15	
Temperature	100 K	
Wavelength	0.71073 Å	
Crystal system	Monoclinic	
Space group	P 21/c	
Unit cell dimensions	a = 13.843(11) Å	α = 90°.
	b = 14.883(13) Å	β = 94.47(2)°.
	c = 7.352(6) Å	γ = 90°.
Volume	1510(2) $Å^3$	
Z	4	
Density (calculated)	2.178 Mg/m^3	
Absorption coefficient	10.092 mm^{-1}	
F(000)	936	
Crystal size	0.3 x 0.22 x 0.1 mm^3	
Theta range for data collection	2.737 to 25.999°.	
Index ranges	-17<=h<=17, -17<=k<=18, -9<=l<=8	
Reflections collected	7218	
Independent reflections	2937 [R(int) = 0.0485]	
Completeness to theta = 25.000°	99.4 %	
Absorption correction	Semi-empirical from equivalents	
Max. and min. transmission	0.1200 and 0.0553	
Refinement method	Full-matrix least-squares on F^2	
Data / restraints / parameters	2937 / 0 / 184	
Goodness-of-fit on F^2	1.015	
Final R indices [I>2sigma(I)]	R1 = 0.0409, wR2 = 0.0912	
R indices (all data)	R1 = 0.0600, wR2 = 0.1002	
Extinction coefficient	n/a	
Largest diff. peak and hole	3.388 and -2.291 e.$Å^{-3}$	

Table S7. Atomic coordinates (x 10^4) and equivalent isotropic displacement parameters ($Å^2$x 10^3) for [($^{t\text{-Bu}}$AQ)$AuCl_2$]. U(eq) is defined as one third of the trace of the orthogonalized U^{ij} tensor.

	x	y	z	U(eq)
Au(1)	6449(1)	9905(1)	5950(1)	15(1)
Cl(1)	5111(2)	9992(1)	7631(3)	20(1)
Cl(2)	6749(2)	11394(1)	6470(4)	23(1)
O(1)	9007(4)	10411(4)	5665(10)	25(2)
N(1)	6320(5)	8556(4)	5576(11)	16(2)
N(2)	7607(5)	9706(4)	4470(10)	15(2)
C(1)	5569(6)	8036(5)	5950(13)	19(2)
C(2)	5635(6)	7097(5)	5828(13)	19(2)
C(3)	6469(6)	6706(5)	5297(14)	22(2)
C(4)	7258(6)	7241(5)	4828(14)	19(2)
C(5)	8142(7)	6905(5)	4245(14)	22(2)
C(6)	8860(6)	7498(6)	3900(14)	22(2)
C(7)	8752(5)	8453(5)	4015(12)	15(2)
C(8)	7885(6)	8807(5)	4531(13)	17(2)
C(9)	7148(5)	8178(5)	4946(13)	14(2)
C(10)	8359(6)	10395(6)	4468(14)	21(2)
C(11)	8298(6)	11025(5)	2812(14)	20(2)
C(12)	7296(7)	11115(6)	1816(14)	26(2)
C(13)	8670(6)	11962(6)	3403(14)	23(2)
C(14)	9003(7)	10605(6)	1524(15)	30(2)

Table S8. Bond lengths [Å] and angles [°] for [($^{t\text{-Bu}}$AQ)$AuCl_2$].

Au(1)-Cl(1)	2.309(3)
Au(1)-Cl(2)	2.281(3)
Au(1)-N(1)	2.033(7)
Au(1)-N(2)	2.028(6)
O(1)-C(10)	1.207(11)
N(1)-C(1)	1.341(10)
N(1)-C(9)	1.388(10)
N(2)-C(8)	1.392(10)
N(2)-C(10)	1.460(10)
C(1)-H(1)	0.9300
C(1)-C(2)	1.404(11)
C(2)-H(2)	0.9300
C(2)-C(3)	1.377(12)
C(3)-H(3)	0.9300
C(3)-C(4)	1.416(12)
C(4)-C(5)	1.418(12)
C(4)-C(9)	1.406(11)
C(5)-H(5)	0.9300
C(5)-C(6)	1.368(12)
C(6)-H(6)	0.9300
C(6)-C(7)	1.432(11)
C(7)-H(7)	0.9300
C(7)-C(8)	1.391(11)
C(8)-C(9)	1.435(11)
C(10)-C(11)	1.535(13)
C(11)-C(12)	1.523(13)
C(11)-C(13)	1.537(11)
C(11)-C(14)	1.543(12)
C(12)-H(12A)	0.9600
C(12)-H(12B)	0.9600
C(12)-H(12C)	0.9600
C(13)-H(13A)	0.9600
C(13)-H(13B)	0.9600
C(13)-H(13C)	0.9600

C(14)-H(14A)	0.9600
C(14)-H(14B)	0.9600
C(14)-H(14C)	0.9600
Cl(2)-Au(1)-Cl(1)	89.96(7)
N(1)-Au(1)-Cl(1)	93.55(19)
N(1)-Au(1)-Cl(2)	174.3(2)
N(2)-Au(1)-Cl(1)	174.79(18)
N(2)-Au(1)-Cl(2)	95.19(19)
N(2)-Au(1)-N(1)	81.3(3)
C(1)-N(1)-Au(1)	127.1(6)
C(1)-N(1)-C(9)	120.6(7)
C(9)-N(1)-Au(1)	112.3(5)
C(8)-N(2)-Au(1)	110.5(5)
C(8)-N(2)-C(10)	118.7(7)
C(10)-N(2)-Au(1)	119.4(5)
N(1)-C(1)-H(1)	119.8
N(1)-C(1)-C(2)	120.3(8)
C(2)-C(1)-H(1)	119.8
C(1)-C(2)-H(2)	120.0
C(3)-C(2)-C(1)	120.0(7)
C(3)-C(2)-H(2)	120.0
C(2)-C(3)-H(3)	119.6
C(2)-C(3)-C(4)	120.7(7)
C(4)-C(3)-H(3)	119.6
C(3)-C(4)-C(5)	125.1(8)
C(9)-C(4)-C(3)	116.9(7)
C(9)-C(4)-C(5)	118.0(8)
C(4)-C(5)-H(5)	120.5
C(6)-C(5)-C(4)	119.0(8)
C(6)-C(5)-H(5)	120.5
C(5)-C(6)-H(6)	118.4
C(5)-C(6)-C(7)	123.3(8)
C(7)-C(6)-H(6)	118.4
C(6)-C(7)-H(7)	120.4
C(8)-C(7)-C(6)	119.2(7)

C(8)-C(7)-H(7)	120.4
N(2)-C(8)-C(9)	115.8(7)
C(7)-C(8)-N(2)	126.7(7)
C(7)-C(8)-C(9)	117.0(7)
N(1)-C(9)-C(4)	121.2(7)
N(1)-C(9)-C(8)	115.2(7)
C(4)-C(9)-C(8)	123.5(7)
O(1)-C(10)-N(2)	120.2(8)
O(1)-C(10)-C(11)	123.9(8)
N(2)-C(10)-C(11)	115.8(8)
C(10)-C(11)-C(13)	109.5(8)
C(10)-C(11)-C(14)	103.9(7)
C(12)-C(11)-C(10)	115.2(7)
C(12)-C(11)-C(13)	109.2(7)
C(12)-C(11)-C(14)	109.6(9)
C(13)-C(11)-C(14)	109.1(7)
C(11)-C(12)-H(12A)	109.5
C(11)-C(12)-H(12B)	109.5
C(11)-C(12)-H(12C)	109.5
H(12A)-C(12)-H(12B)	109.5
H(12A)-C(12)-H(12C)	109.5
H(12B)-C(12)-H(12C)	109.5
C(11)-C(13)-H(13A)	109.5
C(11)-C(13)-H(13B)	109.5
C(11)-C(13)-H(13C)	109.5
H(13A)-C(13)-H(13B)	109.5
H(13A)-C(13)-H(13C)	109.5
H(13B)-C(13)-H(13C)	109.5
C(11)-C(14)-H(14A)	109.5
C(11)-C(14)-H(14B)	109.5
C(11)-C(14)-H(14C)	109.5
H(14A)-C(14)-H(14B)	109.5
H(14A)-C(14)-H(14C)	109.5
H(14B)-C(14)-H(14C)	109.5

Symmetry transformations used to generate equivalent atoms:

Table S9. Anisotropic displacement parameters ($Å^2 x 10^3$) for [($^{t\text{-Bu}}$AQ)$AuCl_2$]. The anisotropic displacement factor exponent takes the form: -2π^2[$h^2 a^{*2}U^{11}$ + ... + 2 h k a* b* U^{12}]

	U^{11}	U^{22}	U^{33}	U^{23}	U^{13}	U^{12}
Au(1)	19(1)	12(1)	15(1)	1(1)	2(1)	0(1)
Cl(1)	23(1)	18(1)	21(1)	1(1)	6(1)	2(1)
Cl(2)	29(1)	13(1)	27(2)	-2(1)	4(1)	-2(1)
O(1)	20(3)	30(3)	24(5)	4(3)	0(3)	-7(2)
N(1)	19(4)	15(3)	14(5)	4(3)	6(3)	-4(2)
N(2)	15(3)	16(3)	15(5)	0(3)	7(3)	2(3)
C(1)	18(4)	28(4)	12(6)	5(4)	0(4)	-2(3)
C(2)	29(5)	17(4)	11(6)	1(3)	3(4)	-8(3)
C(3)	28(5)	14(4)	24(6)	6(3)	-3(4)	0(3)
C(4)	22(5)	20(4)	14(6)	2(3)	1(4)	4(3)
C(5)	30(5)	19(4)	18(6)	-3(4)	5(4)	2(3)
C(6)	18(5)	30(5)	19(6)	2(4)	-2(4)	12(3)
C(7)	12(4)	24(4)	9(5)	2(3)	2(4)	-1(3)
C(8)	26(5)	17(4)	8(5)	4(3)	0(4)	-2(3)
C(9)	10(4)	20(4)	14(6)	1(3)	-2(3)	0(3)
C(10)	21(5)	21(4)	20(6)	2(4)	4(4)	-4(3)
C(11)	22(5)	19(4)	19(6)	-1(4)	8(4)	1(3)
C(12)	35(6)	25(4)	18(6)	7(4)	-2(4)	-7(4)
C(13)	28(5)	25(4)	15(6)	4(4)	0(4)	-5(3)
C(14)	41(6)	26(5)	23(7)	4(4)	8(5)	-3(4)

Table S10. Hydrogen coordinates (x 10^4) and isotropic displacement parameters ($Å^2$x 10^3) for [($^{t\text{-Bu}}$AQ)$AuCl_2$].

	x	y	z	U(eq)
H(1)	5001	8300	6292	23
H(2)	5116	6740	6105	23
H(3)	6515	6083	5246	27
H(5)	8232	6291	4100	27
H(6)	9448	7270	3576	27
H(7)	9256	8832	3746	18
H(12A)	7064	10532	1432	39
H(12B)	7334	11495	767	39
H(12C)	6859	11376	2620	39
H(13A)	8242	12221	4227	34
H(13B)	8691	12340	2349	34
H(13C)	9309	11911	4004	34
H(14A)	9634	10551	2153	45
H(14B)	9040	10982	472	45
H(14C)	8772	10021	1144	45

II) [($^{t\text{-Bu}}$AQ)AuCl$_2$]

B) ^{1}H NMR Spectrum

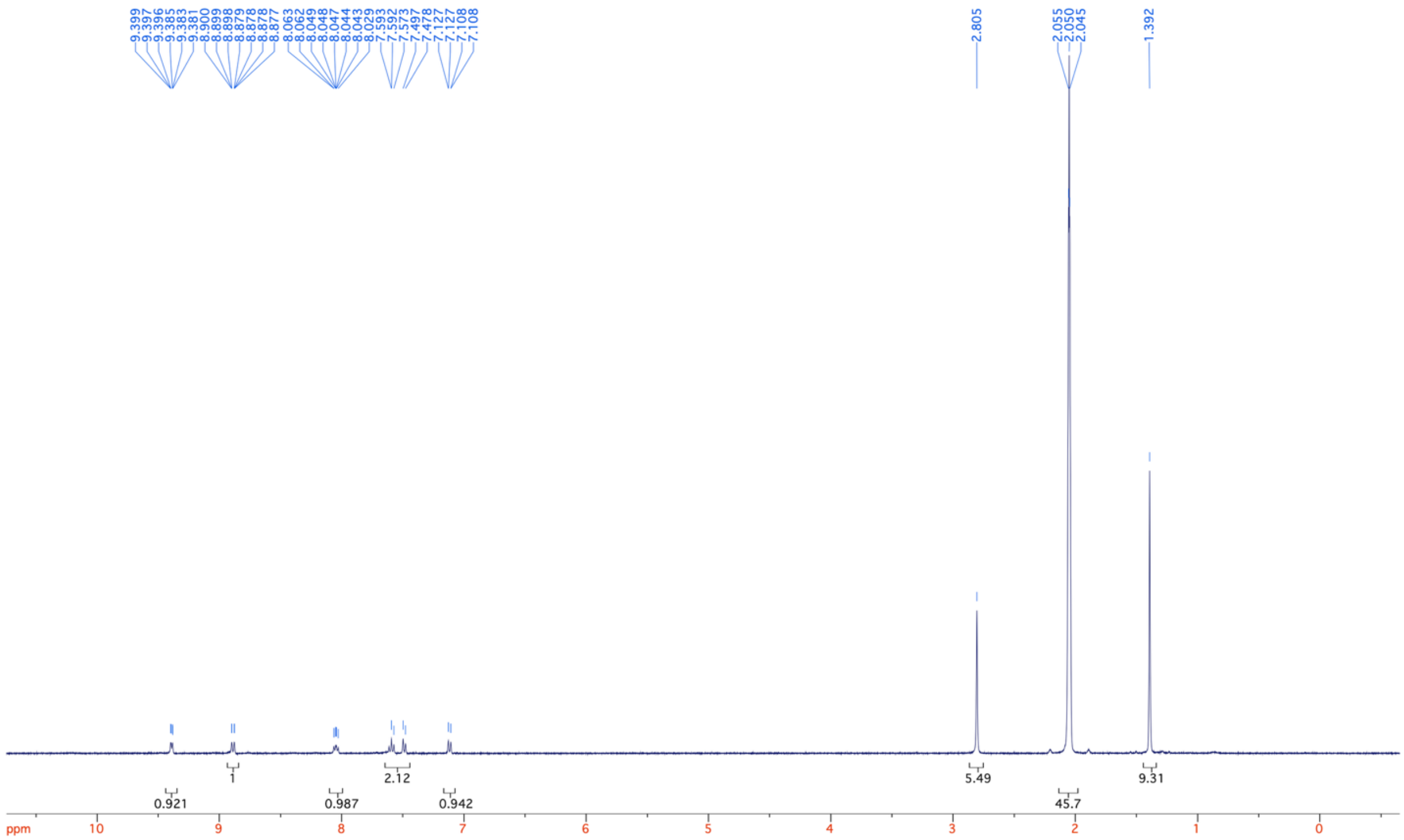

Figure S3. ^{1}H NMR spectrum of [($^{t\text{-Bu}}$AQ)AuCl$_2$] in acetone-d_6.

II) [($^{t\text{-Bu}}$AQ)$AuCl_2$]

C) $^{13}C\{^1H\}$ NMR Spectrum

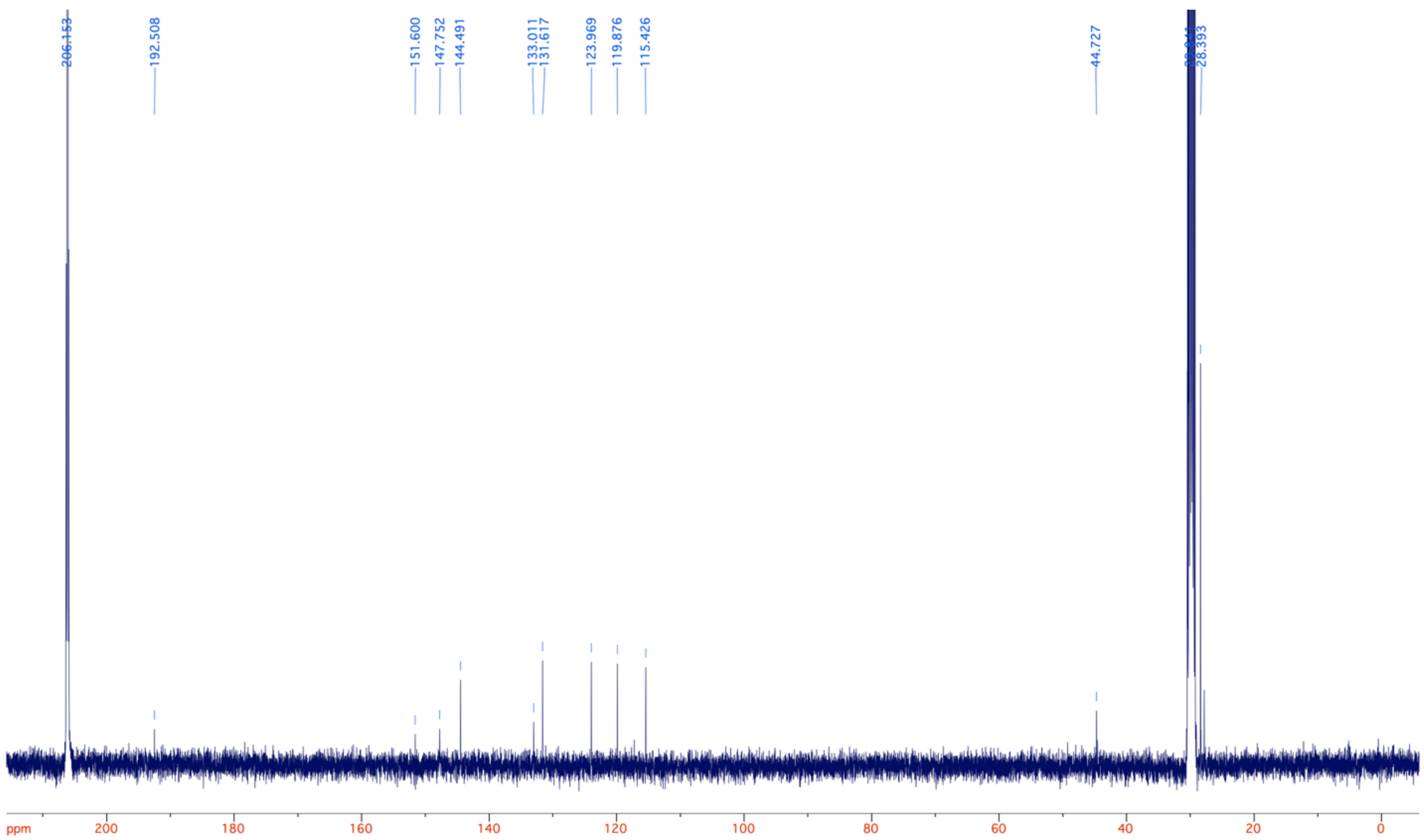

Figure S4. $^{13}C\{^1H\}$ NMR spectrum of [($^{t\text{-Bu}}$AQ)$AuCl_2$] in acetone-d_6.

II) [($^{t\text{-Bu}}$AQ)$AuCl_2$]

D) ^{1}H—^{13}C HSQC NMR Spectrum

Figure S5. ^{1}H—^{13}C NMR spectrum of [($^{t\text{-Bu}}$AQ)$AuCl_2$] in acetone-d_6.

II) [($^{t\text{-}Bu}$AQ)AuCl$_2$]

E) UV-Vis Spectrum

Figure S6. UV-Vis spectrum of 0.1 mM [($^{t\text{-}Bu}$AQ)AuCl$_2$] in CH_2Cl_2.

II) [($^{t\text{-Bu}}$AQ)AuCl$_2$]

F) IR Spectrum

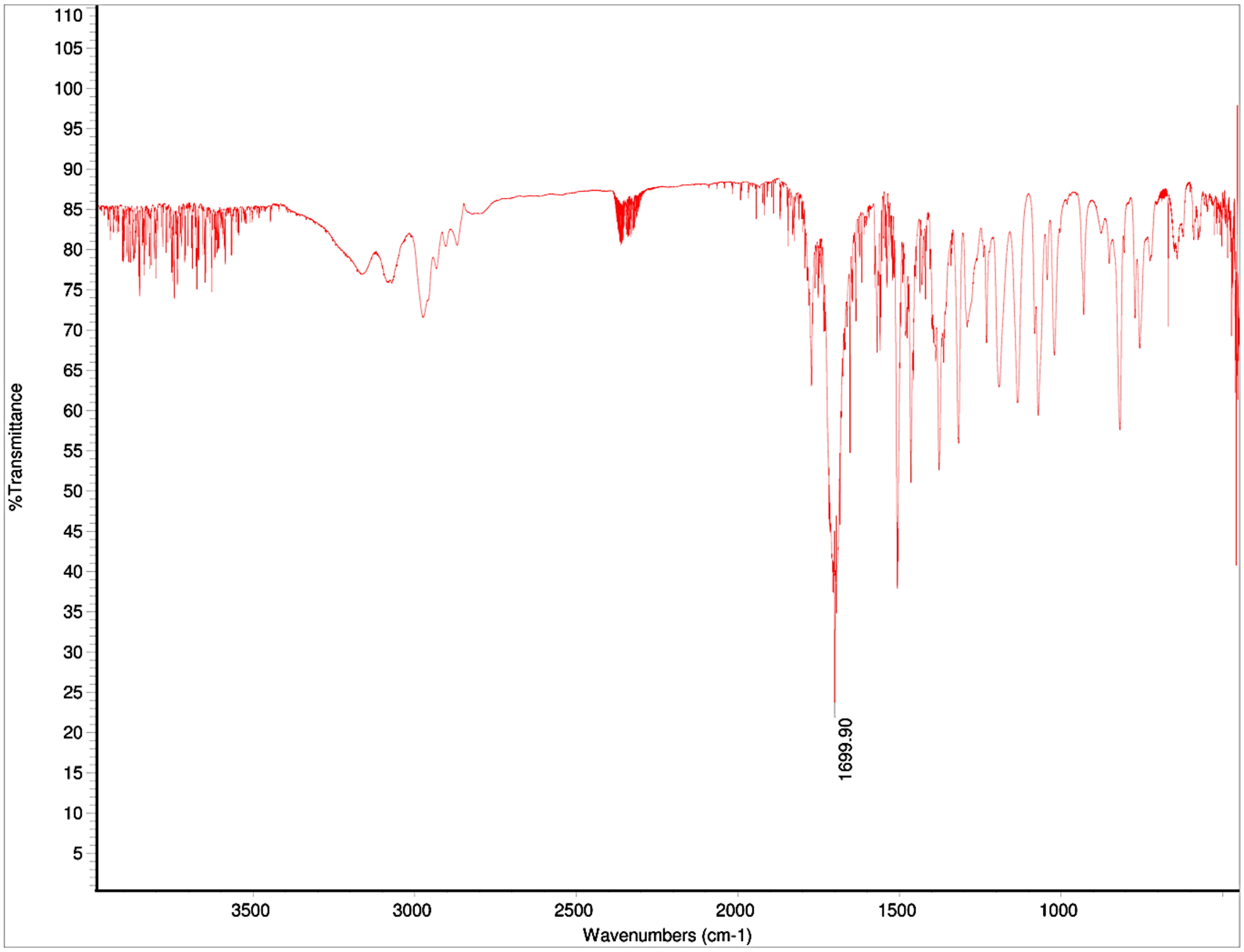

Figure S7. IR spectrum of [($^{t\text{-Bu}}$AQ)AuCl$_2$] on a NaCl plate with the carbonyl stretch labelled at 1699.90 cm^{-1}.

III) [($^{3\text{-Pent}}$AQ)AuCl$_2$]

A) Single Crystal X-ray Diffraction

Table S11. Crystal data and structure refinement for [($^{3\text{-Pent}}$AQ)AuCl$_2$].

Identification code	weinberg01
Empirical formula	$C_{15}H_{17}AuCl_2N_2O$
Formula weight	509.17
Temperature/K	100.0
Crystal system	triclinic
Space group	P-1
a/Å	7.8386(11)
b/Å	9.2402(16)
c/Å	11.468(2)
α/°	86.946(4)
β/°	75.714(4)
γ/°	89.178(4)
Volume/Å^3	803.8(2)
Z	2
ρ_{calc}g/cm^3	2.104
μ/mm^{-1}	9.483
F(000)	484.0
Crystal size/mm^3	0.2 × 0.2 × 0.2
Radiation	MoKα (λ = 0.71073)
2Θ range for data collection/°	4.414 to 56.778
Index ranges	$-10 \leq h \leq 10$, $-12 \leq k \leq 12$, $-15 \leq l \leq 15$
Reflections collected	24086
Independent reflections	4034 [R_{int} = 0.1209, R_{sigma} = 0.0599]
Data/restraints/parameters	4034/0/192
Goodness-of-fit on F^2	1.021
Final R indexes [I>=2σ (I)]	R_1 = 0.0307, wR_2 = 0.0749
Final R indexes [all data]	R_1 = 0.0348, wR_2 = 0.0762
Largest diff. peak/hole / e Å^{-3}	3.27/-2.40

Table S12. Fractional Atomic Coordinates (×10^4) and Equivalent Isotropic Displacement Parameters (Å^2×10^3) for [($^{3\text{-Pent}}$AQ)AuCl$_2$]. U_{eq} is defined as 1/3 of the trace of the orthogonalised U_{IJ} tensor.

**Atom**	***x***	***y***	***z***	**U(eq)**
Au(1)	4594.4(2)	6694.1(2)	5916.7(2)	8.43(7)
Cl(1)	6643.7(17)	7023.5(14)	4122.9(10)	16.8(3)
Cl(2)	2450.2(16)	7331.9(13)	4969(1)	14.9(2)
O(1)	1012(5)	6983(4)	9264(3)	17.2(8)
N(1)	6342(5)	5832(4)	6801(3)	9.8(8)
N(2)	2954(5)	6316(4)	7551(3)	9.2(8)
C(1)	8088(7)	5814(5)	6445(4)	13.5(10)
C(2)	9095(7)	4945(6)	7054(5)	16.8(10)
C(3)	8280(7)	4055(6)	8015(5)	15.8(10)
C(4)	6419(7)	4032(5)	8404(4)	11.4(9)
C(5)	5437(7)	3143(5)	9372(4)	16.4(11)
C(6)	3621(8)	3254(6)	9692(4)	16.6(11)
C(7)	2718(7)	4257(5)	9102(4)	13.1(10)
C(8)	3640(6)	5156(5)	8167(4)	10.0(9)
C(9)	5475(7)	4989(5)	7789(4)	10.7(9)
C(10)	1824(6)	7311(5)	8223(4)	11.4(9)
C(11)	1669(6)	8814(5)	7675(4)	11.7(9)
C(12)	-274(7)	9199(5)	7804(4)	16.3(10)
C(13)	-520(8)	10463(6)	6950(5)	26.4(13)
C(14)	2580(7)	9876(6)	8315(5)	18.5(11)
C(15)	4610(7)	9691(6)	7991(5)	22.8(12)

Table S13. Anisotropic Displacement Parameters ($Å^2 \times 10^3$) for [($^{3\text{-Pent}}$AQ)$AuCl_2$]. The Anisotropic displacement factor exponent takes the form: $-2\pi^2[h^2a^{*2}U_{11}+2hka^*b^*U_{12}+\ldots]$.

**Atom**	**U_{11}**	**U_{22}**	**U_{33}**	**U_{23}**	**U_{13}**	**U_{12}**
Au(1)	9.56(10)	7.11(10)	8.02(10)	-0.32(6)	-1.07(6)	0.39(7)
Cl(1)	16.7(6)	18.5(6)	11.7(5)	0.8(4)	2.4(4)	0.0(5)
Cl(2)	17.7(6)	16.2(6)	12.6(5)	-0.7(4)	-7.5(5)	3.3(5)
O(1)	16.6(18)	17.3(18)	13.6(16)	1.8(14)	3.2(14)	3.5(15)
N(1)	10.8(19)	13(2)	7.3(17)	-3.3(15)	-4.0(15)	2.2(16)
N(2)	13(2)	8.4(18)	4.8(16)	0.9(14)	-0.6(15)	2.3(16)
C(1)	12(2)	14(2)	15(2)	-5.4(19)	-0.9(19)	-2.5(19)
C(2)	10(2)	21(3)	22(3)	-9(2)	-8(2)	1(2)
C(3)	15(3)	15(2)	21(2)	-8(2)	-10(2)	4(2)
C(4)	15(2)	8(2)	13(2)	-4.8(17)	-7.0(19)	1.1(18)
C(5)	27(3)	9(2)	16(2)	-1.5(19)	-10(2)	2(2)
C(6)	27(3)	10(2)	12(2)	0.4(18)	-3(2)	-2(2)
C(7)	13(2)	14(2)	13(2)	-3.9(18)	-3.1(19)	-2.8(19)
C(8)	15(2)	8(2)	9(2)	-4.9(17)	-4.7(18)	2.1(18)
C(9)	18(2)	5(2)	12(2)	-5.3(17)	-7.9(19)	-0.2(18)
C(10)	10(2)	11(2)	14(2)	-0.2(18)	-3.3(18)	0.9(18)
C(11)	13(2)	11(2)	10(2)	-0.1(17)	-0.4(18)	1.3(19)
C(12)	16(3)	11(2)	18(2)	1.0(19)	0(2)	2(2)
C(13)	22(3)	18(3)	37(3)	9(2)	-6(3)	9(2)
C(14)	17(3)	15(2)	26(3)	-5(2)	-8(2)	-2(2)
C(15)	16(3)	22(3)	32(3)	-1(2)	-8(2)	-3(2)

Table S14. Bond Lengths for [($^{3\text{-Pent}}$AQ)$AuCl_2$].

Atom	Atom	Length/Å	Atom	Atom	Length/Å
Au(1)	Cl(1)	2.2850(12)	C(4)	C(5)	1.413(7)
Au(1)	Cl(2)	2.2712(12)	C(4)	C(9)	1.415(6)
Au(1)	N(1)	2.027(4)	C(5)	C(6)	1.383(8)
Au(1)	N(2)	2.011(4)	C(6)	C(7)	1.403(7)
O(1)	C(10)	1.230(6)	C(7)	C(8)	1.380(7)
N(1)	C(1)	1.328(6)	C(8)	C(9)	1.405(7)
N(1)	C(9)	1.375(6)	C(10)	C(11)	1.509(6)
N(2)	C(8)	1.424(6)	C(11)	C(12)	1.532(7)
N(2)	C(10)	1.392(6)	C(11)	C(14)	1.539(7)
C(1)	C(2)	1.396(7)	C(12)	C(13)	1.525(7)
C(2)	C(3)	1.367(7)	C(14)	C(15)	1.551(8)
C(3)	C(4)	1.417(7)			

Table S15. Bond Angles for [($^{3\text{-Pent}}$AQ)$AuCl_2$].

Atom	Atom	Atom	Angle/˚	Atom	Atom	Atom	Angle/˚
Cl(2)	Au(1)	Cl(1)	89.32(5)	C(6)	C(5)	C(4)	119.6(5)
N(1)	Au(1)	Cl(1)	94.09(12)	C(5)	C(6)	C(7)	121.8(5)
N(1)	Au(1)	Cl(2)	171.55(12)	C(8)	C(7)	C(6)	120.0(5)
N(2)	Au(1)	Cl(1)	175.04(11)	C(7)	C(8)	N(2)	127.2(4)
N(2)	Au(1)	Cl(2)	95.50(12)	C(7)	C(8)	C(9)	118.5(4)
N(2)	Au(1)	N(1)	80.97(16)	C(9)	C(8)	N(2)	114.2(4)
C(1)	N(1)	Au(1)	128.6(3)	N(1)	C(9)	C(4)	120.6(4)
C(1)	N(1)	C(9)	120.5(4)	N(1)	C(9)	C(8)	117.1(4)
C(9)	N(1)	Au(1)	110.0(3)	C(8)	C(9)	C(4)	122.2(4)
C(8)	N(2)	Au(1)	108.9(3)	O(1)	C(10)	N(2)	120.3(4)
C(10)	N(2)	Au(1)	126.7(3)	O(1)	C(10)	C(11)	120.6(4)
C(10)	N(2)	C(8)	118.9(4)	N(2)	C(10)	C(11)	119.1(4)
N(1)	C(1)	C(2)	121.4(5)	C(10)	C(11)	C(12)	109.9(4)
C(3)	C(2)	C(1)	119.9(5)	C(10)	C(11)	C(14)	108.0(4)
C(2)	C(3)	C(4)	120.1(5)	C(12)	C(11)	C(14)	111.8(4)
C(5)	C(4)	C(3)	125.0(5)	C(13)	C(12)	C(11)	112.4(4)
C(5)	C(4)	C(9)	117.6(5)	C(11)	C(14)	C(15)	112.5(4)
C(9)	C(4)	C(3)	117.4(5)				

Table S16. Torsion Angles for [($^{3\text{-Pent}}$AQ)$AuCl_2$].

A	B	C	D	Angle/°	A	B	C	D	Angle/°
Au(1)	N(1)	C(1)	C(2)	-167.8(4)	C(3)	C(4)	C(9)	N(1)	4.3(6)
Au(1)	N(1)	C(9)	C(4)	166.6(3)	C(3)	C(4)	C(9)	C(8)	-173.9(4)
Au(1)	N(1)	C(9)	C(8)	-15.1(5)	C(4)	C(5)	C(6)	C(7)	-1.8(7)
Au(1)	N(2)	C(8)	C(7)	-157.3(4)	C(5)	C(4)	C(9)	N(1)	-176.7(4)
Au(1)	N(2)	C(8)	C(9)	25.8(5)	C(5)	C(4)	C(9)	C(8)	5.0(7)
Au(1)	N(2)	C(10)	O(1)	-172.8(4)	C(5)	C(6)	C(7)	C(8)	0.7(7)
Au(1)	N(2)	C(10)	C(11)	4.7(6)	C(6)	C(7)	C(8)	N(2)	-173.6(4)
O(1)	C(10)	C(11)	C(12)	-53.1(6)	C(6)	C(7)	C(8)	C(9)	3.2(7)
O(1)	C(10)	C(11)	C(14)	69.1(6)	C(7)	C(8)	C(9)	N(1)	175.6(4)
N(1)	C(1)	C(2)	C(3)	2.1(7)	C(7)	C(8)	C(9)	C(4)	-6.1(7)
N(2)	C(8)	C(9)	N(1)	-7.2(6)	C(8)	N(2)	C(10)	O(1)	-21.9(7)
N(2)	C(8)	C(9)	C(4)	171.1(4)	C(8)	N(2)	C(10)	C(11)	155.6(4)
N(2)	C(10)	C(11)	C(12)	129.4(5)	C(9)	N(1)	C(1)	C(2)	0.1(7)
N(2)	C(10)	C(11)	C(14)	-108.4(5)	C(9)	C(4)	C(5)	C(6)	-1.1(7)
C(1)	N(1)	C(9)	C(4)	-3.3(7)	C(10)	N(2)	C(8)	C(7)	47.1(7)
C(1)	N(1)	C(9)	C(8)	174.9(4)	C(10)	N(2)	C(8)	C(9)	-129.8(5)
C(1)	C(2)	C(3)	C(4)	-1.0(7)	C(10)	C(11)	C(12)	C(13)	-162.1(4)
C(2)	C(3)	C(4)	C(5)	179.0(5)	C(10)	C(11)	C(14)	C(15)	70.1(5)
C(2)	C(3)	C(4)	C(9)	-2.1(7)	C(12)	C(11)	C(14)	C(15)	-168.9(4)
C(3)	C(4)	C(5)	C(6)	177.9(5)	C(14)	C(11)	C(12)	C(13)	78.0(5)

Table S17. Hydrogen Atom Coordinates (Å×10^4) and Isotropic Displacement Parameters (Å^2×10^3) for [($^{3\text{-Pent}}$AQ)$AuCl_2$].

Atom	*x*	*y*	*z*	U(eq)
H(1)	8663	6404	5762	16
H(2)	10344	4972	6801	20
H(3)	8964	3451	8422	19
H(5)	6020	2474	9800	20
H(6)	2968	2635	10329	20
H(7)	1471	4317	9347	16
H(11)	2287	8838	6800	14
H(12A)	-902	8341	7639	20
H(12B)	-806	9453	8645	20
H(13A)	-43	10201	6116	40
H(13B)	102	11315	7109	40
H(13C)	-1777	10685	7078	40
H(14A)	2121	9722	9197	22
H(14B)	2288	10881	8088	22
H(15A)	5109	10127	7182	34
H(15B)	4904	8657	8008	34
H(15C)	5098	10172	8577	34

III) [($^{3\text{-Pent}}$AQ)AuCl$_2$]

B) ^{1}H NMR Spectrum

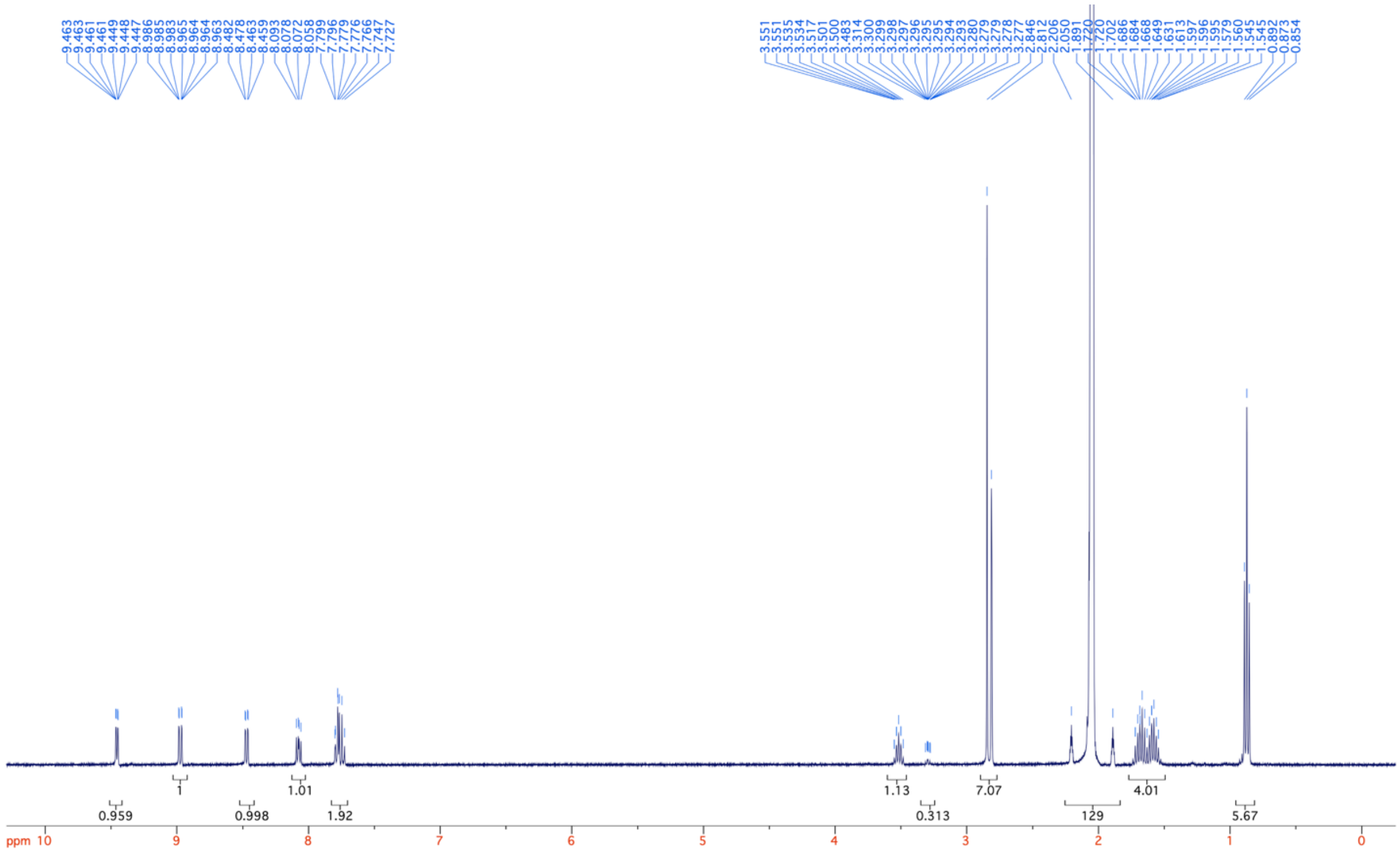

Figure S8. ^{1}H NMR spectrum of [($^{3\text{-Pent}}$AQ)AuCl$_2$] in acetone-d_6.

III) [($^{3\text{-Pent}}$AQ)AuCl$_2$]

C) $^{13}C\{^1H\}$ NMR Spectrum

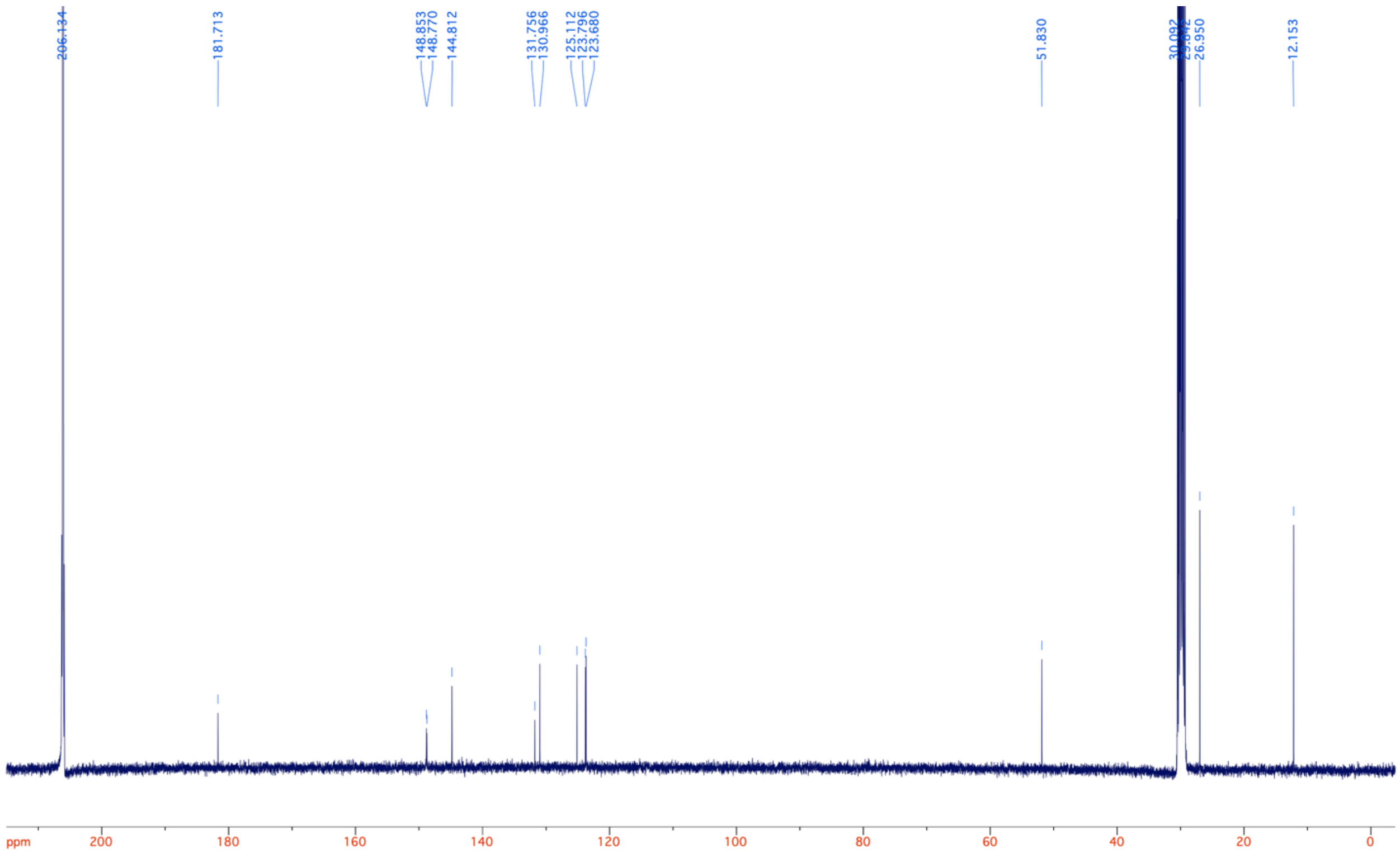

Figure S9. $^{13}C\{^1H\}$ NMR spectrum of [($^{3\text{-Pent}}$AQ)AuCl$_2$] in acetone-d_6.

III) [($^{3\text{-Pent}}$AQ)AuCl$_2$]

D) ^{1}H—^{13}C HSQC NMR Spectrum

Figure S10. ^{1}H—^{13}C NMR spectrum of [($^{3\text{-Pent}}$AQ)AuCl$_2$] in acetone-d_6.

III) $[(^{3\text{-}Pent}AQ)AuCl_2]$

E) UV-Vis Spectrum

Figure S11. UV-Vis spectrum of 0.1 mM $[(^{3\text{-}Pent}AQ)AuCl_2]$ in CH_2Cl_2.

III) [($^{3\text{-Pent}}$AQ)AuCl$_2$]

F) IR Spectrum

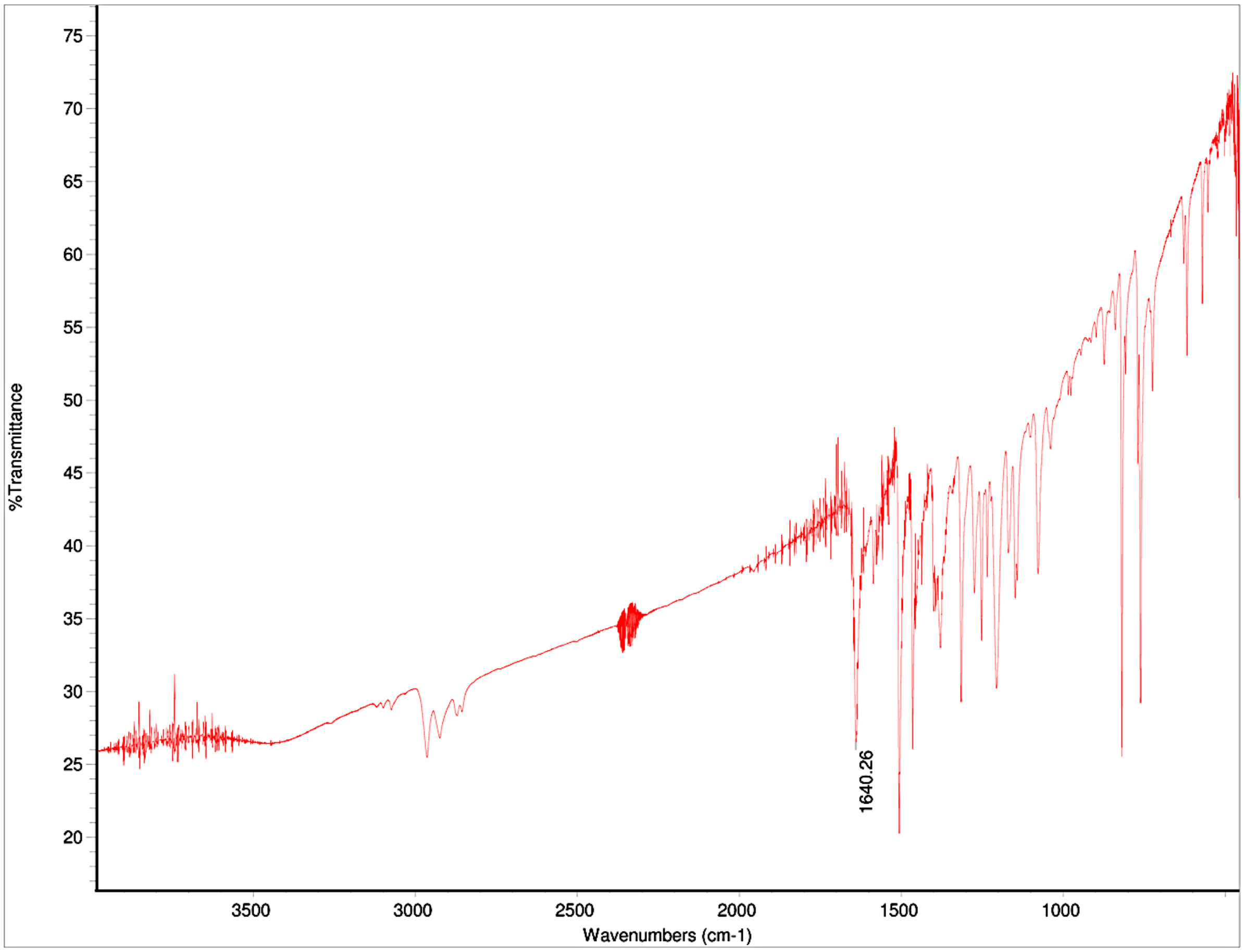

Figure S12. IR Spectrum of [($^{3\text{-Pent}}$AQ)AuCl$_2$] on a NaCl plate with the carbonyl stretch labelled at 1640.26 cm^{-1}.
